# Does the introduction of an infliximab biosimilar always result in savings for hospitals? A descriptive study using real-world data

**DOI:** 10.1186/s13561-024-00507-5

**Published:** 2024-04-29

**Authors:** Marko Krstic, Jean-Christophe Devaud, Farshid Sadeghipour, Joachim Marti

**Affiliations:** 1 Service of Pharmacy, Lausanne University Hospital and University of Lausanne, 1011 Lausanne, Switzerland; 2 Center for Research and Innovation in Clinical Pharmaceutical Sciences, Lausanne University Hospital, University of Lausanne, 1011 Lausanne, Switzerland; 3 Institute of Pharmaceutical Sciences of Western Switzerland, University of Geneva, University of Lausanne, 1206 Geneva, Switzerland; 4 School of Pharmaceutical Sciences, Department of Hospital Pharmacy, University of Geneva, Geneva, 1206 Switzerland; 5 Faculty of Biology and Medicine, University of Lausanne, 1005 Lausanne, Switzerland; 6 Center for Primary Care and Public Health (Unisanté), University of Lausanne, DESS, Health Economics Unit, 1010 Lausanne, Switzerland

**Keywords:** TNF-α inhibitors, Biosimilars, Non-medical switching, Health resource utilization, Real-world data, Infliximab, CT-P13, Hospital formulary, Savings

## Abstract

**Background:**

Biosimilars are biologic drugs that have the potential to increase the efficiency of healthcare spending and curb drug-related cost increases. However, their introduction into hospital formularies through initiatives such as non-medical switching must be carefully orchestrated so as not to cause treatment discontinuation or result in increased health resource utilization, such as additional visits or laboratory tests, among others. This retrospective cohort study aims to assess the impact of the introduction of CT-P13 on the healthcare expenditures of patients who were treated with originator infliximab or CT-P13.

**Methods:**

Gastroenterology, immunoallergology and rheumatology patients treated between September 2017 and December 2020 at a university hospital in Western Switzerland were included and divided into seven cohorts, based on their treatment pathway (i.e., use and discontinuation of CT-P13 and/or originator infliximab). Costs in Swiss francs were obtained from the hospital's cost accounting department and length of stay was extracted from inpatient records. Comparisons of costs and length of stay between cohorts were calculated by bootstrapping.

**Results:**

Sixty immunoallergology, 84 rheumatology and 114 gastroenterology patients were included. Inpatient and outpatient costs averaged (sd) CHF 1,611 (1,020) per hospital day and CHF 4,991 (6,931) per infusion, respectively. The mean (sd) length of stay was 20 (28) days. Although immunoallergology and rheumatology patients had higher average costs than gastroenterology patients, differences in costs and length of stay were not formally explained by treatment pathway. Differences in health resource utilization were marginal.

**Conclusions:**

The introduction of CT-P13 and the disruption of patient treatment management were not associated with differences in average outpatient and inpatient costs and length of stay, in contrast to the results reported in the rest of the literature. Future research should focus on the cost-effectiveness of non-medical switching policies and the potential benefits for patients.

**Supplementary Information:**

The online version contains supplementary material available at 10.1186/s13561-024-00507-5.

## Background

Biological drugs (i.e. biologics) are drugs composed of complex molecules usually produced by biotechnology using living organisms or their cells [1, 2]. They have improved the treatment of common conditions such as diabetes or hemophilia, added new therapeutic options in oncology, and revolutionized the management of a wide range of autoimmune diseases for which therapeutic options were often limited in effectiveness or non-existent [3–7]. Given the much higher costs of research and development, pharmaceutical companies have been charging higher prices for biologics than for chemical drugs [8]. The craze for biologics has been putting pressure on government health systems and has limited the accessibility of these innovative products to a large number of patients, especially in emerging countries [9–11].

Biosimilars are biologics that are highly similar to their reference biologics (i.e., originators) with which they share few or no clinical differences [12, 13]. Even though clinicians are increasingly comfortable with the use of biosimilars in biologic-naïve patients, some concerns remain about switching patients who are stable on the originator, for reasons other than efficacy or side effects (i.e., non-medical switching [NMS]) [3, 5, 14–19].

Biosimilars can only be marketed after the originator's patent has expired and at purchase prices that are 10% to 40% lower than the originator's [12, 20, 21]. Biosimilars thus ensure access to care for treated patients while sparing the paying parties [21–24]. The present study focuses on the case of infliximab and its biosimilar, CT-P13.

Infliximab and CT-P13 are murine chimeric monoclonal antibodies that act as tumor necrosis factor alpha (TNF-⍺) antagonists, also known as biologic disease-modifying antirheumatic drugs (bDMARDs). They are used to treat a wide range of inflammatory autoimmune diseases refractory to conventional immunosuppressive treatments [3–5, 25–27]. To date, switching from OI to CT-P13 has not posed major issues. Indeed, their equivalence has been extensively demonstrated in rigorous randomized clinical trials [28–33], assessed in reviews that have gathered a plethora of real-world studies [34, 35] and is supported by the U. S. Food and Drug Administration and the European Medicines Agency [8, 27, 36, 37]. CT-P13 was infliximab’s first biosimilar approved by the EU in 2013 and was granted market access in Switzerland in 2016 [27, 38]. Although no political and/or financial incentives have emerged in Switzerland to promote biosimilars use since their first appearance in 2009, hospitals have been gradually introducing biosimilars into their formularies due to the expected savings on drug purchasing costs [39].

However, the economic impact of an NMS from an originator biologic to its biosimilar is still subject to debate and additional real-world studies are required [8]. Two systematic reviews identified a handful of studies on biosimilar NMS, the majority of which were conference abstracts [40, 41]. Modeling studies aside, both reviews emphasized that too many economic evaluations were based solely on drug purchase prices and that future evaluations should include health resource utilization (HRU) in their assessment to fully capture the economic implications of an originator-to-biosimilar NMS. At first sight, the costs of training medical staff, laboratory tests or administrative procedures, for example, may seem marginal or non-existent compared to the savings made on the basis of the biosimilar price alone. Yet this may not be the case if other costs are considered, such as the cost of switching to a second or a third treatment, additional outpatient visits or the cost of additional hospital stays. A recent real-world study in a large tertiary hospital in Western Switzerland reported an improperly implemented NMS strategy from OI to CT-P13 and an exceptional CT-P13 treatment discontinuation rate, again raising the question of the economic impact of such a cost control strategy [8].

The aim of this retrospective cohort study is therefore to analyze differences in outpatient and inpatient costs and length of stay between patients who initiated CT-P13 or switched from OI, who discontinued treatment or who continued to take the same biologic, at the above-mentioned hospital.

## Methods

### Study population, setting and comparators

**Fig. 1:**
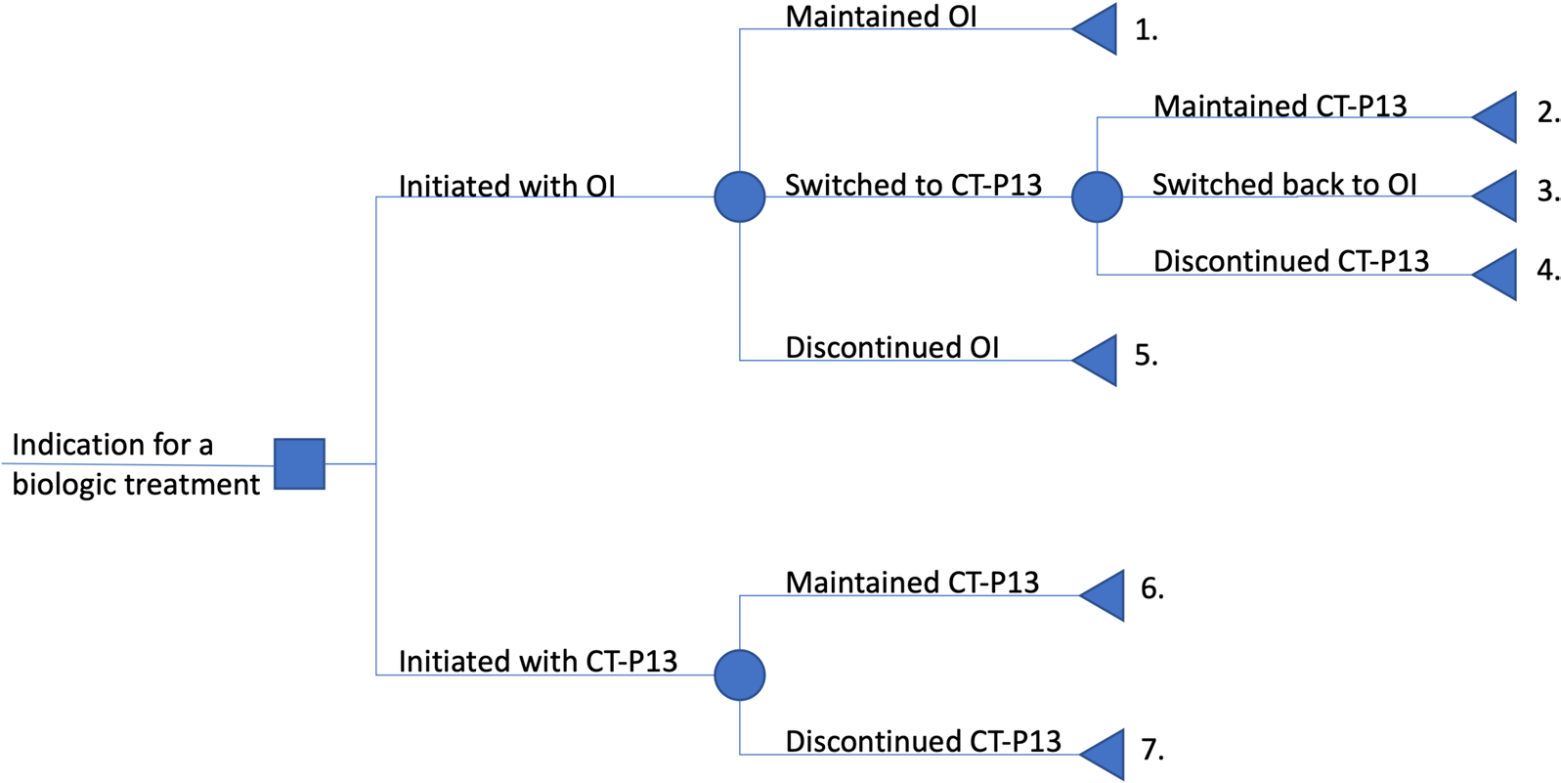
Decision tree describing the seven cohorts compared. *OI = originator infliximab*

The study population consisted of patients with various inflammatory autoimmune diseases who were part of a previous retrospective study examining the reasons for discontinuation of the infliximab biosimilar CT-P13 in a university hospital in Western Switzerland [8]. In summary, CT-P13 was introduced in the hospital's formulary in late September 2017 with no formal communication or education protocol for healthcare professionals or patients. Furthermore, the use of CT-P13 and OI was only passively monitored without analyzing individual cases, whether patients switched from OI to CT-P13, started CT-P13 treatment, or stopped their treatment. Out of 320 eligible patients, the previous study included 156 patients and found a 37% overall rate of CT-P13 discontinuation after one year. In the present study, only the following patients were not included: 1) patients who received OI or CT-P13 primarily for autoimmune symptoms caused by their underlying oncology treatment; 2) patients who received only one infusion of either OI or CT-P13; and 3) patients who declined consent. Two authors independently included the patients, and two different authors allocated them in the cohorts presented on Fig. 1.

This study adopted a hospital perspective because OI and CT-P13 are biologic drugs that are primarily administered in the hospital, either in an outpatient or inpatient setting.

### Measurement and valuation of resources and costs

For each year, actual costs were extracted by the cost accounting department at the closing of the accounts in March of the following year, in Swiss francs (CHF) (e.g., the final costs for the year 2017 were closed in March 2018). Each patient’s costs were detailed into 37 expenditure items (see Additional file 1), providing information on HRU. Costs were not updated to 2022 or converted to U.S. dollars because exchange rates for CHF 1 varied on average between $1.02 and $1.07 from 2017 to 2020 [42].

Regarding inpatients cohorts, length of stay (LOS) in days was used to refine the analysis. LOS was calculated automatically from inpatient administrative data and was not valued in a monetary unit.

### Analytics and assumptions

As each patient had multiple outpatient and inpatient stays, their costs, number of outpatient visits (*i.e.*, number of infusions) and LOS were aggregated by the sum. Cost per hospital day (HD) and cost per infusion at the patient level were obtained by dividing inpatient costs by LOS, and outpatient costs by number of infusions. Continuous (*i.e.*, costs, LOS and age) and categorical (*i.e.*, sex and disease category) variables were reported using descriptive statistics: mean (standard deviation), median (interquartile range), and number (percentage).

A comprehensive examination of the data's distribution and dispersion was conducted prior to the main analysis. Pairwise comparisons were adjusted using the Holm-Bonferroni method. Formal comparisons of mean costs and LOS between cohorts were performed using random sampling with replacement (*i.e.*, bootstrapping) with 5 000 bootstrap replicates and percentile confidence intervals at 2.5% and 97.5%. Visual inspection of the bootstrap replicates from each cohort was conducted using histograms. Following a top-down approach, significant differences in mean costs per infusion or per HD between cohorts guided our analyses at the expenditure item level. Pairwise comparisons of expenditure items with fewer than 2 respective patients were dropped because bootstrap analyses could not be calculated. Bootstrap percentile analyses provide so-called “conservative” wide confidence intervals, which, by reducing type I error (i.e., false positives), address the priority in our analysis of accounting for the limited number of patients in the cohorts and the uncertainty surrounding our findings [24, 43].

General linear models (GLMs) were used to estimate how costs and LOS varied by age, sex and disease category and were compared using the Likelihood ratio test. GLMs were computed using a Gamma distribution and a log link to account for positive skewness of both cost and LOS data. The alpha significance level for all tests was set at 0.05, and all analyses were performed using R (version 4.2.0, R Foundation for Statistical Computing, Vienna, Austria) and are available in Additional file 1 [44].

This study was written using relevant points from the latest Consolidated Health Economic Evaluation Reporting Standards 2022 (CHEERS) Statement [45].

## Results

### Patients’ characteristics

Out of 320 eligible patients, 258 (81%) were included and distributed in the seven cohorts. Sixty-two patients were not included: 28 (45%) received originator infliximab (OI) or CT-P13 as part of an underlying oncology treatment; 23 (37%) patients did not consent to the re-use of their data, 7 (11%) patients received only one OI infusion, and 4 (6%) patients received only one CT-P13 infusion. Descriptive statistics regarding patients’ age, sex and disease category are reported in Table 1.

**Table 1:** Characteristics of included patients. Percentages are rounded and are for guidance only

**Cohort**	**Sex** *n* **tot. (%F)**	**Median Age** **years (IQR)**	**Disease category** *n* **(%)**		
			**GAS**	**IMM**	**RHE**
*Inpatients and outpatients*	*258 (54)*	*42 (28)*	*114 (44)*	*60 (23)*	*84 (33)*
Started OI and maintained OI	78 (52)	45 (30)	31 (40)	28 (36)	19 (25)
Started CT-P13 and maintained CT-P13	43 (51)	36 (25)	17 (40)	14 (33)	12 (28)
Started OI and discontinued OI	19 (79)	34 (25)	11 (58)	1 (5)	7 (37)
Started CT-P13 and discontinued CT-P13	32 (56)	32 (19)	18 (56)	6 (19)	8 (25)
Switched from OI to CT-P13 and maintained CT-P13	54 (46)	48 (32)	25 (46)	6 (11)	23 (43)
Switched from OI to CT-P13 and discontinued CT-P13	16 (50)	39 (28)	7 (44)	2 (13)	7 (44)
Switched from OI to CT-P13 and switched back to OI	16 (75)	54 (13)	5 (31)	3 (19)	8 (50)

*%F* percentage of female patients, *GAS* Gastroenterology, *IMM* Immunoallergology, *IQR* Inter-quartile range, *OI* Originator infliximab, *tot* total number of patients

The cohort “switched from OI to CT-P13 and switched back to OI” (54 years old, 13) was older than both cohorts “started OI and discontinued OI” (34 years old, 25), and “started CT-P13 and discontinued CT-P13” (36 years old, 25) (*p* < 0.01). Regarding the sex ratio by cohort, there were more female patients in “started OI and discontinued OI” (*n* = 15, 79%) compared to “switched from OI to CT-P13 and maintained CT-P13” (*n* = 25, 46%) (*p* < 0.03).

### Costs

**Fig. 2:**
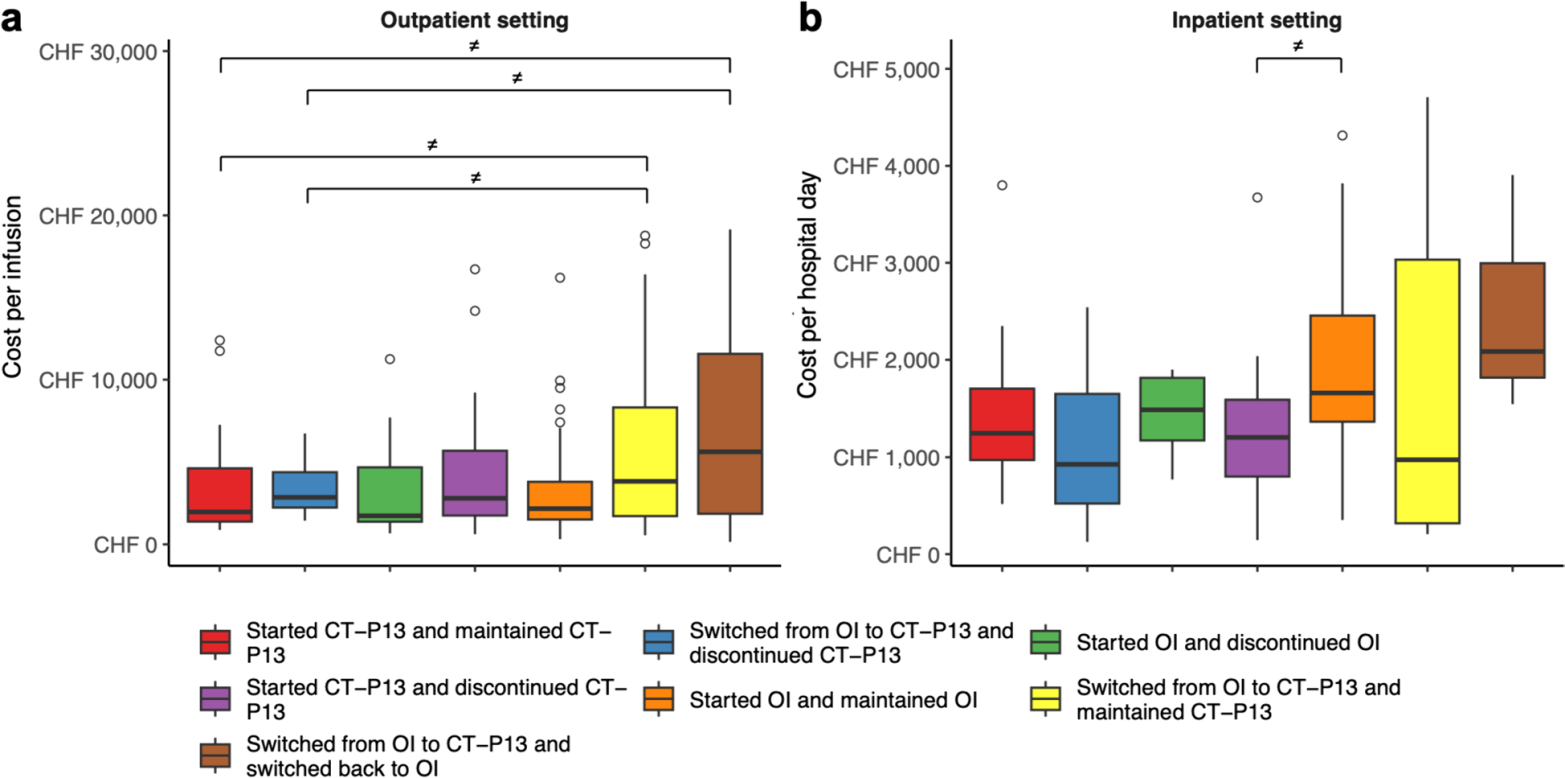
Box plots of (**a**) outpatient and (**b**) inpatient costs, per cohort. Outliers were represented by empty circles and hidden over CHF 30 000. *OI = originator infliximab; ≠ significant difference in mean costs*

Outpatient costs averaged (sd) CHF 4 991 (6 931) per infusion, and inpatient costs averaged CHF 1 611 (1 020) per hospital day (HD). Cohorts that differed in outpatient and/or inpatient costs are presented in Fig. 2 and described with the rest of the cohorts in Table 2. Pairwise bootstrap analyses of outpatient costs showed that both cohorts “Started CT-P13 and maintained CT-P13” and “Switched from OI to CT-P13 and discontinued CT-P13” had lower mean costs per infusion than cohorts “Switched from OI to CT-P13 and maintained CT-P13” and “Switched from OI to CT-P13 and switched back to OI”. The same analysis for inpatient costs reported that cohort “Started CT-P13 and discontinued CT-P13” had a lower mean cost per HD than cohort “Started OI and maintained OI”.

**Table 2:** Details on average costs per infusion and per hospital day, respectively, for outpatients and inpatients. Percentages are rounded and are for guidance only

**Cohort**	**Patients** *n* **(%)**	**Costs** mean **(sd) [CHF/inf./HD]**	**Costs** mean** (**PCI**)** **[CHF/inf./HD]**
*Outpatients*	*255 (100)*	*4 991 (6 931)*	*—*
Started OI and maintained OI	76 (30)	5 351 (10 225)	(3 458 ; 7 942)
Started CT-P13 and maintained CT-P13	43 (17)	3 317 (2 681)	(2 566 ; 4 135)
Started OI and discontinued OI	19 (7)	3 276 (3 083)	(2 044 ; 4 732)
Started CT-P13 and discontinued CT-P13	31 (12)	5 379 (6 387)	(3 431 ; 7 839)
Switched from OI to CT-P13 and maintained CT-P13	54 (21)	5 966 (5 573)	(4 562 ; 7 489)
Switched from OI to CT-P13 and discontinued CT-P13	16 (6)	3 407 (1 612)	(2 657 ; 4 205)
Switched from OI to CT-P13 and switched back to OI	16 (6)	7 359 (6 350)	(4 462 ; 10 413)
*Inpatients*	*94 (100)*	*1 611 (1 020)*	*—*
Started OI and maintained OI	27 (29)	2 033 (1 096)	(1 641 ; 2 440)
Started CT-P13 and maintained CT-P13	22 (23)	1 457 (747)	(1 176 ; 1 781)
Started OI and discontinued OI	6 (6)	1 439 (453)	(1 088 ; 1 743)
Started CT-P13 and discontinued CT-P13	21 (22)	1 267 (767)	(968 ; 1 612)
Switched from OI to CT-P13 and maintained CT-P13	8 (9)	1 727 (1 748)	(671 ; 2 917)
Switched from OI to CT-P13 and discontinued CT-P13	7 (7)	1 134 (855)	(585 ; 1 764)
Switched from OI to CT-P13 and switched back to OI	3 (3)	2 513 (1 236)	(1 547 ; 3 905)

*HD* Hospital day, *inf.* Infusion, *OI* Originator infliximab, *PCI* Percentile confidence intervals, *sd* standard deviation

**Fig. 3:**
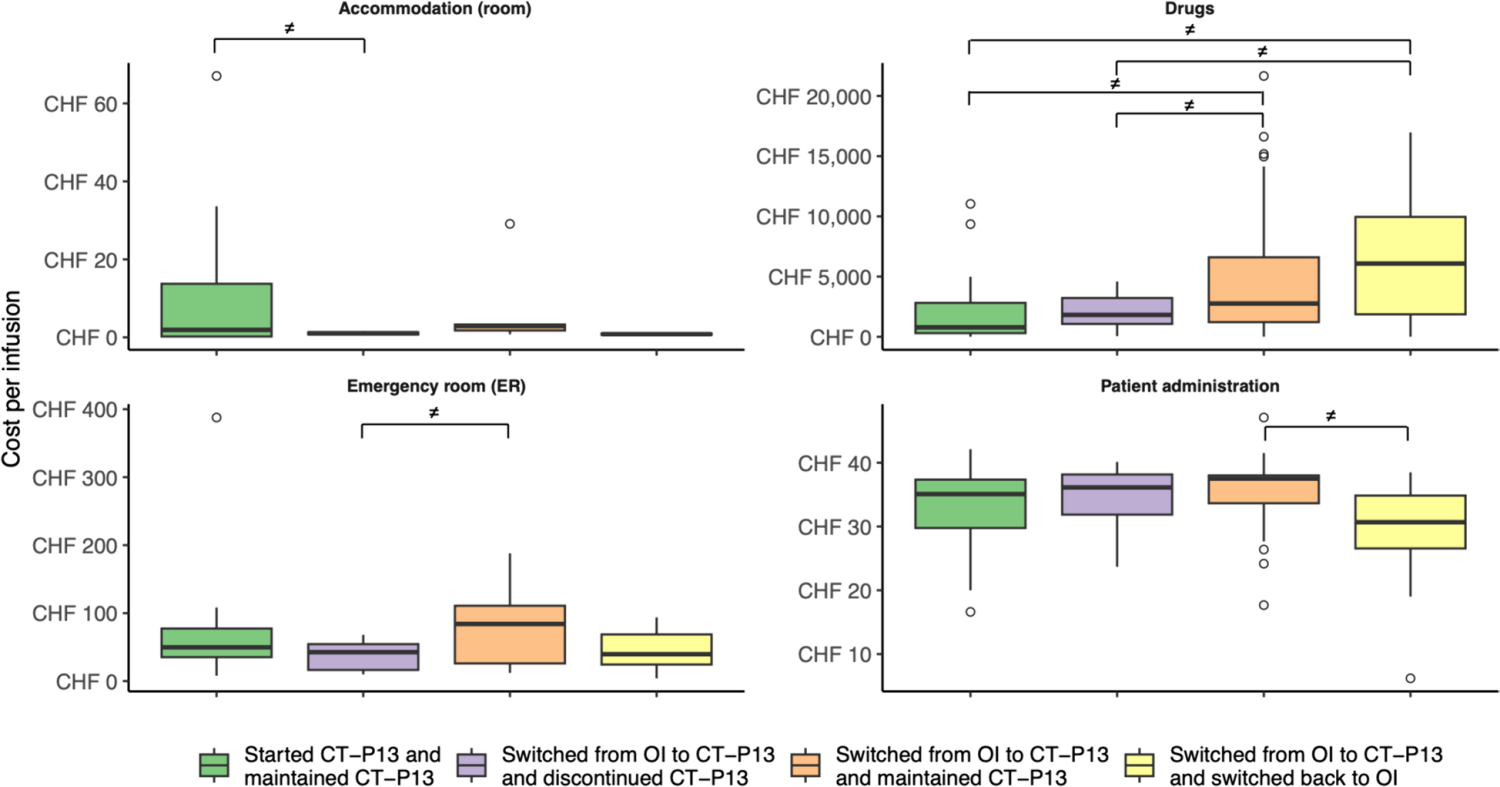
Box plots of outpatient expenditure items for which costs differed significantly based on bootstrap analyses. The cost axis was adjusted according to each expenditure item. Outliers were represented by empty circles*. OI* = *originator infliximab; ≠ significant difference in mean costs*

In terms of expenditure items, 4 outpatient items differed in terms of average costs per infusion, with “Drugs” being the most important (Fig. 3). As for the inpatient setting, only item “Medical staff (intermediate care unit, *i.e.*, IMCU)” was significantly higher for cohort “Started OI and maintained OI” (CHF 11.34 – CHF 32.22 per HD vs CHF 32.41 – CHF 79.61 per HD). It should be noted that several expenditure items could not be compared because none of the patients in cohort “Started CT-P13 and discontinued CT-P13” received the corresponding benefits, including: “Blood and blood products”, “Intensive care units (ICU) and IMCU”, “Medical staff (ICU)”, and “Nuclear medicine and radiation oncology”.

Multivariable analysis suggested that both outpatient and inpatient cost were affected by disease category *(p* < 0.01). In the outpatient setting, rheumatology (RHE) patients had higher mean costs per infusion than both gastroenterology (GAS) and immunoallergology (IMM) patients, while in the inpatient setting, both RHE and IMM patients had higher mean cost per HD than GAS patients. In the latter case, GLMs also reported that age was positively associated with costs (p < 0.01). However, there was a small correlation between age and disease categories where the youngest patients associated with the lowest inpatient costs were predominantly GAS patients, thus isolating age as a confounder. The models’ parameters and scatterplots are available in Additional file 1.

### Length of inpatient stay

**Fig. 4:**
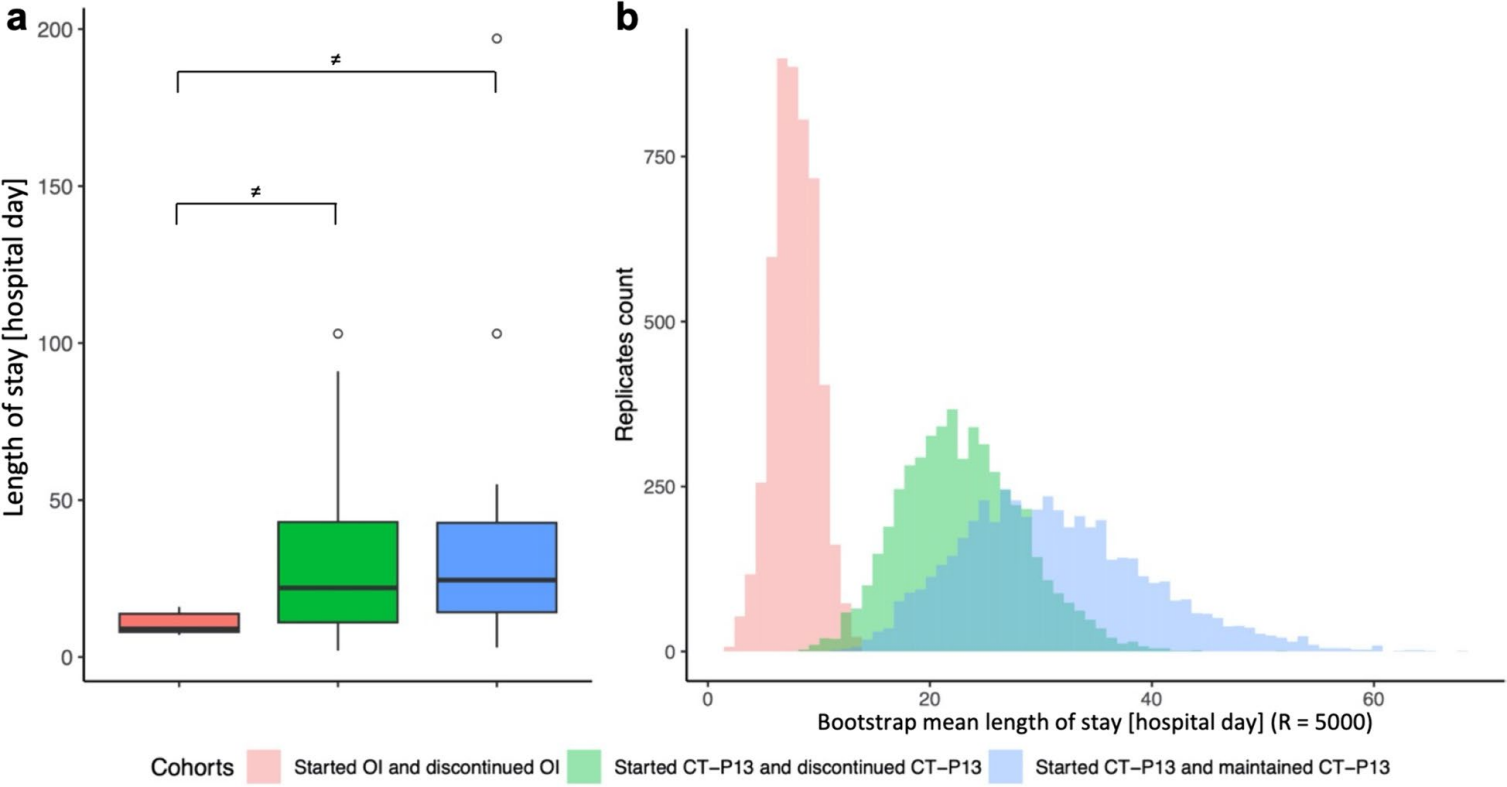
(**a**) Boxplot of inpatients’ length of stay, by cohort. (**n**) Histogram of the 5 000 bootstrap replicates of inpatients’ mean LOS, by cohort. *OI* = *originator infliximab; ≠ bootstrap percentile confidence intervals do not overlap*

The mean (sd) LOS per inpatient was 20 days [28]. Cohorts that differed significantly are presented in Fig. 4 and described with the rest of the cohorts in Table 3. Thus, cohort “Started CT-P13 and discontinued CT-P13” had significantly lower LOS than both cohorts “Started OI and discontinued OI” and “Started CT-P13 and maintained CT-P13”. Multivariable analysis suggested that neither sex, age nor disease categories affected inpatient LOS.

**Table 3:** Details on inpatients’ length of stay. Percentages are rounded and are for guidance only

**Cohort**	**Patients** *n* **(%)**	**LOS** **mean** **(sd) [HD]**	**LOS** **mean (PCI) [HD]**
*Inpatients*	*94 (100)*	*20 (28)*	—
Started OI and maintained OI	27 (29)	12 (13)	(8 ; 18)
Started CT-P13 and maintained CT-P13	22 (23)	31 (41)	(17 ; 50)
Started OI and discontinued OI	6 (6)	8 (5)	(4 ; 12)
Started CT-P13 and discontinued CT-P13	21 (22)	23 (26)	(13 ; 35)
Switched from OI to CT-P13 and maintained CT-P13	8 (9)	8 (13)	(2 ; 17)
Switched from OI to CT-P13 and discontinued CT-P13	7 (7)	12 (12)	(4 ; 21)
Switched from OI to CT-P13 and switched back to OI	3 (3)	62 (42)	(15 ; 95)

*HD* Hospital day, *LOS* Length of stay, *OI* Originator infliximab, *PCI* Percentile confidence intervals, *sd* standard deviation

## Discussion

From a hospital perspective, this study sought to determine and better understand whether the introduction of the CT-P13 biosimilar resulted in differences in outpatient and inpatient expenditures or LOS. As the equivalence of OI and CT-P13 has been well established in the literature, the discussion will not cover differences in terms of safety, efficacy, or immunogenicity.

Regarding outpatient cohorts, “Started-CT-P13 and maintained CT-P13” and “Switched from OI to CT-P13 and discontinued CT-P13” had a lower average cost per infusion than cohorts “Switched from OI to CT-P13 and maintained CT-P13” and “Switched from OI to CT-P13 and switched back to OI”. In terms of expenditure items, “Drugs” was the primary cause of cost differences, while differences in health resource utilization (HRU) were negligible (*i.e.*, “Accommodation (room)”, “Emergency room” and “Patient administration”). However, given that “Drugs” included all drugs administered to patients, it is not possible to relate the differences in costs in this item to the use of OI and/or CT-P13 or to argue that most of the observed cost differences are solely due to lower list prices of CT-P13 (-8.5% in 2022 in Switzerland) [46]. Furthermore, our results do not allow us to explain the observed differences in outpatient costs by a combination of higher OI purchase prices and expenditures caused by disruption in patient treatment management. Indeed, although cohort “Started-CT-P13 and maintained CT-P13” had the lowest average cost per infusion and cohort “Switched from OI to CT-P13 and switched back to OI had the highest average cost per infusion, the results from cohorts “Switched from OI to CT-P13 and discontinued CT-P13” and “Switched from OI to CT-P13 and maintained CT-P13” did not satisfy the hypothesis that cohorts that primarily used CT-P13 and/or did not switch would have a lower mean cost per infusion. Given that disease category was highlighted as a significant independent variable for outpatient costs, it appears that differences in average cost per infusion were primarily due to patients’ disease, regardless of their use of OI or CT-P13 or changes in treatment management.

Among the inpatient cohorts, only cohort “Started OI and maintained OI”, which used exclusively OI, had a significantly higher mean cost per HD than cohort “Started CT-P13 and discontinued CT-P13”, which initiated treatment with the biosimilar and subsequently discontinued it. Although there is a gap between the list prices of OI and CT-P13, this difference was not identified in the “Drugs” item, mainly because inpatient expenditures are compensated for by a Swiss Diagnostic Related Groups (SwissDRG), the Swiss hospital case classification and compensation system [47, 48]. Therefore, only expenses outside the scope of the corresponding SwissDRGs would be eligible for additional compensation. No significant differences were identified among the other items, which would have corresponded to differences in HRU. Interestingly, some patients in the cohort “Started OI and maintained OI” were admitted to the ICU, which undoubtedly contributed to the overall costs of the cohort. Fortunately for the patients, but unfortunately for the analyses, no patients in cohort “Started CT-P13 and discontinued CT-P13” required ICU management, and thus it was not possible to identify a corresponding difference in HRU. Nevertheless, it is highly unlikely that the use of OI or CT-P13, or the initiation or discontinuation of these biologics, could have led to worsened patient outcomes, although it may have contributed to the total inpatient costs generated. Once again, in the GLMs that examined the relationship between costs and independent variables, disease category emerged as a significant variable. It is therefore likely, as was the case for the outpatient setting, that cost differences were due to patients' diseases rather than their therapeutic management and/or substitution with a biosimilar. In fact, cohort “Started OI and discontinued OI” was composed mostly of RHE and IMM patients, who were associated with higher costs than GAS patients, according to the GLMs.

Regarding LOS, which is inevitably a predictor of hospitalization costs and a variable of great interest to hospitals that are compensated by a DRG system, the GLMs revealed no variables that could explain the differences between the two pairs of cohorts identified as different. If one were only interested in the patients' treatment pathway, one would have to conclude that patients treated with CT-P13 had longer hospital stays than patients on OI, whether they continued their treatment or switched to another treatment. Although visual inspection of the histogram of the sampled replicates of hospital LOS for cohort “Started OI and discontinued OI” was satisfactory, this cohort included only 6 patients. Thus, the conclusions of the bootstrap analyses could be challenged to conclude that there were, in fact, no significant differences between the cohorts in LOS, which is consistent with the GLM analyses.

### Impact of non-medical switching from originator infliximab to biosimilar CT-P13

Although some cohorts had higher costs than others, these differences could only be identified using analyses based on bootstrap resampling. Indeed, given the relatively small number of patients, the right-skewed distribution of data, and the need to compare cost averages and not their medians, it was not possible to use “standard” statistical methods such as Student's *t*-test to determine whether two cohorts were different or not [49]. This study used the percentile bootstrap method, which has the advantage of reducing the risk of Type I error (*i.e.*, false positives) by providing wide, and therefore, conservative confidence intervals (CIs) [43]. However, by using this type of CIs we may have missed some differences in HRU reported in two recent systematic reviews on the non-medical switch (NMS) from biologics to biosimilars [40, 41]. For example, several studies reported increased HRU after an NMS from OI to CT-P13 [50, 51]. In our opinion, these differences did not elude us; we did not analyze them. This is due to our top-down approach that was different from these studies that only compared expenditures items without consideration of total costs. If we had compared only the expenditure items, we would certainly have found additional differences in HRU between the seven cohorts. On a side note, the lack of difference in costs also contrasts with the savings reported by conference abstracts found in the literature [52–54]. In this case, these pilot studies only considered the purchase prices of OI and CT-P13, which is insufficient for a comprehensive evaluation [40].

Based on our results, and given the statistical considerations mentioned, it is unlikely that the introduction of CT-P13 is the cause of the cost differences identified between the cohorts. Additionally, this means that even without rigorous implementation and monitoring, CT-P13 introduction did not result in a significant increase in HRU in both outpatient and inpatient settings. Therefore, prioritizing a biosimilar with a lower purchase price is a dominant strategy for the patient, the hospital, and the healthcare system. In the first case, in the outpatient setting, the patient will have a lower direct contribution to healthcare costs and will therefore reduce the total sum of his or her direct payments in the form of healthcare premiums and co-payments, as well as reducing the costs borne by the community. In the second case, in the hospital, the risk of exceeding the allocated SwissDRG lump sums due to the use of an expensive specialty that does not have a special additional remuneration is reduced. Finally, from a health system perspective, the reduction in opportunity cost of biosimilar use frees up resources to be allocated to other services. Hence, our findings indicate that initiation or switching of patients to the CT-P13 biosimilar in the hospital setting should be given consideration, regardless of their medical history or future clinical course.

### Limitations

Our results must be considered with several limitations. Firstly, our results are subject to some selection bias since patients were not included in the different cohorts randomly, but on the basis of observable variables. It is therefore possible that unobservable elements explain the few differences in costs described above. In addition, there is a possible classification bias where a patient was allocated to the wrong cohort, thus reducing the robustness of our conclusions. However, this bias was sufficiently controlled by the separate allocation of patients by two authors. Secondly, the retrospective use of billing data may be hampered by some degree of data deficiency. Indeed, some services provided at the hospital are not necessarily billed, for *e.g.*, when the industry provides the first months of treatment or when physicians obtain the drugs outside the usual hospital pharmacy circuit. Although we consider this data deficiency to be limited or negligible, its random occurrence prevented us from formally linking the outpatient visits and inpatient stays used by health staff to those used by cost accounting to bill them. In an ideal environment, each OI and CT-P13 infusion would have been captured and billed electronically with a unique stay number, which would have allowed us to identify precisely when patients started, changed, or interrupted their treatment, and allowed us to further refine the construction of the patient cohorts. Thirdly, the use of socio-economic or health severity variables as independent variables would have improved the quality of our multivariate analysis and highlighted their role in the costs of these biological treatments. As in the previous retrospective study, we were confronted with the unavailability of data or the impossibility of extracting it automatically and systematically [8]. Finally, our results must be interpreted taking into account the relatively small number of patients in our study, which was limited to the patient population of only one of the five university hospitals in Switzerland. Combining data from other centers to increase the sample size was hampered by constraints related to data sources and the landscape of healthcare digitization in Switzerland.

## Conclusions

This descriptive cost study is one of the few analyses in the literature to use real-world data to examine the differences in outpatient and inpatient costs and length of stay between patients treated with CT-P13 and original infliximab (OI). Although CT-P13 has lower list and wholesale prices than OI, these differences were not reflected in overall outpatient or inpatient costs. Similarly, changes in therapeutic management caused by the implementation of a non-medical substitution strategy from OI to CT-P13 were not related to increased drug costs or healthcare resource utilization. This study contrasts with previous publications, which have reported mixed results regarding the impact of CT-P13 on hospital costs and supports the routine introduction of biosimilars in the hospital setting.

### Supplementary Information

**Supplementary Material 1.**

## Data Availability

The datasets generated during and/or analyzed during the current study are available from the corresponding author on reasonable request.

## Abbreviations

bDMARDS: Biologic disease-modifying antirheumatic drugs
CHEERS: Consolidated Health Economic Evaluation Reporting Standards
CHF: Swiss francs
CIs: Confidence intervals
GAS: Gastroenterology
GLMs: General linear models
HD: Hospital day
HRU: Health resource utilization
ICU: Intensive care unit
IMCU: Intermediate care unit
IMM: Immunoallergology
LOS: Length of stay
NMS: Non-medical switch
RHE: Rheumatology
SwissDRG: Swiss Diagnosis Related Groups
TNF-α: Tumor necrosis factor alpha
