# Supplementary material for: Does the introduction of an infliximab biosimilar always result in savings for hospitals? A descriptive study using real-world data
Source: Health Econ Rev. 2024 Apr 29;14:31. doi: 10.1186/s13561-024-00507-5 (PMC11059762; doi:10.1186/s13561-024-00507-5)
Supplement: Supplementary file 1 — Supplementary Material 1. [file 13561_2024_507_MOESM1_ESM.docx]

**Does the introduction of an infliximab biosimilar always result in savings for hospitals? A descriptive study using real-world data.**

*Health economics review*

Marko KRSTIC, MS ^a, b, c, d^, Jean-Christophe DEVAUD, PhD ^b, c^, Farshid SADEGHIPOUR, PhD ^a, b, c, d^, Joachim MARTI, PhD ^e, f^

^a^ Institute of Pharmaceutical Sciences of Western Switzerland, University of Geneva, University of Lausanne,
 1206 Geneva, Switzerland

^b^ Service of Pharmacy, Lausanne University Hospital and University of Lausanne, 1011 Lausanne, Switzerland

^c^ Center for Research and Innovation in Clinical Pharmaceutical Sciences, Lausanne University Hospital and
 University of Lausanne, 1011 Lausanne, Switzerland

^d^ School of Pharmaceutical Sciences, Department of Hospital Pharmacy, University of Geneva,1206 Geneva,
 Switzerland

^e^ Faculty of Biology and Medicine, University of Lausanne, 1005 Lausanne, Switzerland

^f^ Center for Primary Care and Public Health (Unisanté), University of Lausanne, DESS, Health Economics Unit,
 1010 Lausanne, Switzerland

Corresponding author address: Farshid SADEGHIPOUR, PhD, Service de pharmacie, Bâtiment hospitalier, Rue du Bugnon 46, 1003 Lausanne, [Farshid.Sadeghipour@unil.ch](mailto:Farshid.Sadeghipour@unil.ch), +41 79 556 32 30, ORCID ID: 0000-0003-0817-5393

**Table of contents**

[I. Disease of the included patients 2](#_Toc130924860)

[II. Classification of the included patients 2](#_Toc130924861)

[III. Expense items provided by the hospital’s cost accounting department 3](#_Toc130924862)

[IV. Conversion rates 4](#_Toc130924863)

[V. Changes in patient treatment pathways 4](#_Toc130924864)

[VI. Statistical methods 4](#_Toc130924865)

[VII. Outcome analysis 5](#_Toc130924866)

[VII.I. Outpatient costs 5](#_Toc130924867)

[VII.I.I. Normality tests and plots 6](#_Toc130924868)

[VII.I.II. Outpatient costs per cost account 7](#_Toc130924869)

[VII.I.III. Comparison of means by bootstrap analysis 11](#_Toc130924870)

[VII.I.IV. Multivariable analysis on costs by generalized linear model (GLM) 12](#_Toc130924871)

[VII.II. Inpatient costs 13](#_Toc130924872)

[VII.II.I. Normality tests and plots 14](#_Toc130924873)

[VII.II.II. Comparison of means by bootstrap analysis 14](#_Toc130924874)

[VII.II.III. Inpatient costs per cost account 15](#_Toc130924875)

[VII.II.IV. Multivariable analysis on costs by generalized linear model (GLM) 17](#_Toc130924876)

[VII.III. Length of stay (LOS) 18](#_Toc130924877)

[VII.III.I. Normality tests and plots 18](#_Toc130924878)

[VII.III.II. Comparison of means by bootstrap analysis 19](#_Toc130924879)

[VII.III.III. Multivariable analysis on costs by generalized linear model (GLM) 19](#_Toc130924880)

[VIII. Variable analysis 20](#_Toc130924881)

[VIII.I. Age 20](#_Toc130924882)

[VIII.I.I. Mean and median comparisons 20](#_Toc130924883)

[VIII.I.II. Normality tests and plots 21](#_Toc130924884)

[VIII.II. Sex 22](#_Toc130924885)

# Diseases of the included patients

**Table I** Details on the disease of the included patients

| Disease category | Disease | Patients *n* (%) |
| --- | --- | --- |
| Gastroenterology | Crohn's Disease | 72 (28 %) |
|  | Ulcerative colitis | 42 (16 %) |
| Immunoallergology | Behçet's disease | 17 (7 %) |
|  | Celiac disease | 1 (0.4 %) |
|  | Cogan's syndrome | 2 (1 %) |
|  | Hidrosadenitis | 6 (2 %) |
|  | IL-17 receptor A and adenosine deaminase 2 deficiency | 1 (0.4 %) |
|  | Polychondritis | 3 (1 %) |
|  | Psoriasis | 4 (2 %) |
|  | Pyoderma gangrenosum | 2 (1 %) |
|  | Sarcoidosis | 19 (7 %) |
|  | Takayasu's Disease | 2 (1 %) |
|  | Uveitis | 3 (1 %) |
| Rheumatology | Ankylosing spondylitis | 38 (15 %) |
|  | Juvenile arthritis | 11 (4 %) |
|  | Psoriatic arthritis | 13 (5 %) |
|  | Rheumatoid arthritis | 22 (9 %) |

# Classification of the included patients

#### Gastroenterology

**Figure I.** Classification of patients included according to their cohort in the gastroenterology group (*n* = 114). *OI = originator infliximab*

#### Immunoallergology

**Figure II.** Classification of patients included according to their cohort in the immunoallergology group (*n* = 60). *OI = originator infliximab*

#### Rheumatology

**Figure III.** Classification of patients included according to their cohort in the rheumatology group (*n* = 84). *OI = originator infliximab*

# Expenditures items provided by the hospital’s cost accounting department

**Table II** Expense item labels provided by the cost accounting department.

| Item N° | Label |
| --- | --- |
| 10 | Patient Administration |
| 20 | Operating Rooms |
| 21 | Cardiac Catheterization Laboratory |
| 23 | Anesthesia |
| 24 | Intensive Care (ICU) and Intermediate Care (IMCU) HotelUnits |
| 25 | Emergency Room (ER) |
| 26 | Medical Imaging Process |
| 27 | Delivery room |
| 28 | Nuclear medicine and radiation oncology |
| 29 | Laboratory |
| 31 | Medical staff in general |
| 32 | Physiotherapy |
| 33 | Occupational therapy |
| 34 | Speech therapy |
| 35 | Non-medical therapies and counseling |
| 36 | Medical diagnosis and therapy |
| 38 | Intermediate Care Units (IMCU) |
| 39 | Care |
| 40 | Psychology |
| 41 | Hotel room service |
| 42 | Hotel kitchen |
| 44 | Other service providers |
| 45 | Pathology |
| 311 | Medical staff operating room activity |
| 312 | Medical staff working in the cardiology room |
| 313 | Medical staff in the intensive care unit (ICU) |
| 314 | Medical staff in intermediate care |
| 315 | Medical staff working in the emergency room (ER) |
| 316 | Medical staff working in the delivery room |
| 317 | Medical staff working in the endoscopy and functional exploration room |
| 380 | Physicians' fees, hospital physicians |
| 400 | Drugs |
| 401 | Equipment, instruments, utensils, textiles |
| 402 | Blood and blood products |
| 404 | Equipment, instruments, utensils, textiles |
| 405 | Medical, diagnostic and therapeutic services of third parties |
| 480 | Patient transport provided by third parties |

# Conversion rates

**Table III** US Dollar – Swiss Franc Exchange Rate^[[1]](#footnote-1)^

| Year | Average closing price |
| --- | --- |
| 2017 | 1.0160 |
| 2018 | 1.0224 |
| 2019 | 1.0062 |
| 2020 | 1.0663 |

# Changes in patient treatment pathways

**Figure IV**. Changes in patient treatment pathways after originator infliximab (OI) or CT-P13 initiation

# Statistical methods

**Table II** Information about the statistical tests used to compare the variables of interest

| Statistic | Purpose | Type | Variable | Additional information |
| --- | --- | --- | --- | --- |
| Analysis of variance (ANOVA) | Equality of means | Parametric | Costs, age, LOS | — |
| Bootstrap resampling | Equality of means | Non-parametric | Costs, age, LOS | For this study, percentile confidence intervals where computed. |
| One_way ANOVA | Equality of means | Parametric | Costs, age, LOS | — |
| Student's *t* test | Equality of means | Parametric | Costs, age, LOS | Holm-Bonferroni’s post-hoc adjustment for multiple pairwise comparisons |
| Kruskal-Wallis test by ranks | Equality of medians | Non-parametric | Costs, age, LOS | — |
| Wilcoxon rank sum test | Equality of medians | Non-parametric | Costs, age, LOS | Holm-Bonferroni’s post-hoc adjustment for multiple pairwise comparisons |
| 2-sample test | Equality of proportions | Non-parametric | Sex | — |
| Fisher's exact test | Equality of proportions | Non-parametric | Sex | Used for small sample sizes |
| Bartlett's test | Equality of variances | Parametric | Costs, age, LOS | Very sensitive to deviations from normality. |
| Fligner-Killeen's test | Equality of variances | Non-parametric | Costs, age, LOS | A very robust alternative against deviations from normality. |
| Levene's test | Equality of variances | Parametric | Costs, age, LOS | A robust alternative to the Bartlett test that is less sensitive to normality deviations. |
| Likelihood ratio test | Goodness of fit | — | Costs, LOS | — |
| Kolmogorov-Smirnov test | Normality of distribution | Non-parametric | Costs, age, LOS | — |
| Shapiro-Wilk normality test | Normality of distribution | Non-parametric | Costs, age, LOS | — |
| *LOS = Length of inpatient stay* | | | | |

# Outcome analysis

## Outpatient costs

**Table IV** Details on outpatient costs and comparison of variances and means by cohort

| Patients  (n) | Total  [CHF] | Mean (sd)  [CHF] | Median (IQR)  [CHF] | max, min  [CHF] | Range  [CHF] | Bartlett  [*p* value] | Levene  [*p* value] | Fligner  [*p* value] | ANOVA  [*p* value] | ANOVA_u  [*p* value] |
| --- | --- | --- | --- | --- | --- | --- | --- | --- | --- | --- |
| 255 | CHF 1 272 727 | CHF 4,991 (6,931) | CHF 2 528 (4 231) | CHF 77 275, CHF 148 | CHF 77 127 | **< 2.20E-16** | 0.26 | **4.27E-05** | 0.26 | **0.01** |
| *ANOVA = Analysis of variance; ANOVA_U = One-way ANOVA or analysis of variance for samples whose variances are not necessarily assumed to be equal; Bartlett = Bartlett's test; Fligner = Fligner-Killeen test; IQR = inter-quartile range; Levene = Levene's test; sd = standard deviation* | | | | | | | | | | |

**Table V** Details on mean and median outpatient costs by cohort, normality assessment of the data was performed using the Shapiro-Wilk normality test and the Kolmogorov-Smirnov test for samples with *n* < 50 patients and *n* > 50 patients, respectively.

| Cohort | patients  (n) | Total  [CHF] | Mean (sd)  [CHF] | Median (IQR)  [CHF] | max, min  [CHF] | Range  [CHF] | Shapiro  [*p* value] | Kolmogorov  [*p* value] |
| --- | --- | --- | --- | --- | --- | --- | --- | --- |
| Started OI and maintained OI | 76 | 406 683 | 5,351 (10,225) | 2 182 (2 431) | 77 275, 312 | 76 964 | — | **2.16E-07** |
| Started CT-P13 and maintained CT-P13 | 43 | 142 625 | 3,317 (2,681) | 1 971 (3 241) | 12 392, 877 | 11 515 | **2.12E-06** | — |
| Started OI and discontinued OI | 19 | 62 235 | 3,276 (3,083) | 1 738 (3 307) | 11 251, 666 | 10 585 | **3.47E-04** | — |
| Started CT-P13 and discontinued CT-P13 | 31 | 166 758 | 5,379 (6,387) | 2 805 (3 941) | 26 099, 615 | 25 484 | **6.61E-07** | — |
| Switched from OI to CT-P13 and maintained CT-P13 | 54 | 322 166 | 5,966 (5,573) | 3 833 (6 615) | 23 697, 549 | 23 148 | — | 0.05 |
| Switched from OI to CT-P13 and discontinued CT-P13 | 16 | 54 516 | 3,407 (1,612) | 2 860 (2 150) | 6 749, 1 441 | 5 308 | 0.28 | — |
| Switched from OI to CT-P13 and switched back to OI | 16 | 117 745 | 7,359 (6,350) | 5 632 (9 708) | 19 147, 148 | 18 999 | 0.06 | — |
| *IQR = inter-quartile range; Kolmogorov = Asymptotic one-sample Kolmogorov-Smirnov test; OI = Originator infliximab; sd = standard deviation; Shapiro = Shapiro-Wilk normality test* | | | | | | | | |

### Normality tests and plots

#### Shapiro-Wilk normality test for all included patients (n samples < 50) - for information purposes only.

W = 0. 0.54599, *p*-value = < 2.2e-16 🡪 *outpatient costs* does not follow a normal distribution

#### Asymptotic one-sample Kolmogorov-Smirnov test all included patients (*n* samples ≥ 50) :

D = 0.25294, p-value = 1.354e-14 🡪 *outpatient costs* does not follow a normal distribution


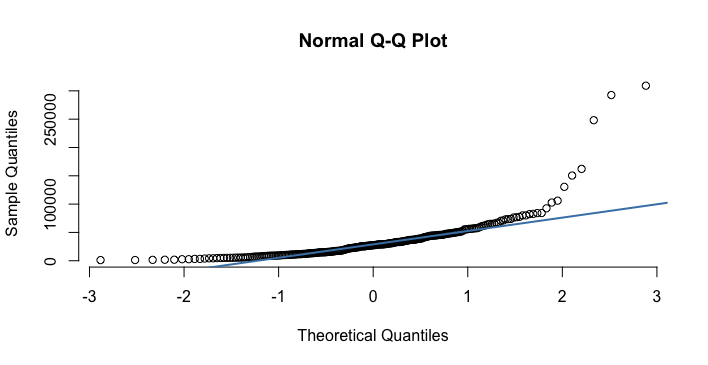


**Figure V.** Quantile-Quantile plot of outpatient costs (left) (*n* = 255)

**Figure VI.** Box plot of outpatient costs (center) (*n* = 255)

**Figure VII.** Box plots of outpatient costs, by cohort

**Figure VIII.** Density plots of outpatient costs, by cohort

### Outpatient costs per cost account

**Table VI** Details on mean and median outpatient costs by cost accounts for the cohort “Started CT-P13 and maintained CT-P13”

| Cost account | Patients (n) | Total [CHF] | Mean (sd) [CHF] | Median (IQR) [CHF] | max, min [CHF] | Range [CHF] |
| --- | --- | --- | --- | --- | --- | --- |
| Accommodation (kitchen) | 1 | 1 | 1 (–) | 1 (–) | 1, 1 | 0 |
| Accommodation (room) | 11 | 133 | 12 (21) | 2 (14) | 67, 0 | 67 |
| Anaesthesia | 14 | 1363 | 97 (155) | 29 (61) | 559, 5 | 553 |
| Blood and blood products | 2 | 123 | 61 (30) | 61 (21) | 82, 40 | 42 |
| Care | 42 | 13,985 | 333 (273) | 215 (341) | 1,190, 27 | 1164 |
| Drugs | 43 | 76,972 | 1,790 (2373) | 784 (2512) | 11,035, 0 | 11035 |
| Emergency room (ER) | 19 | 1317 | 69 (82) | 50 (42) | 388, 8 | 380 |
| Equipment, instruments, utensils, textiles | 43 | 1112 | 26 (29) | 13 (27) | 109, 1 | 108 |
| Laboratory | 43 | 8471 | 197 (139) | 150 (167) | 676, 51 | 625 |
| Medical and therapeutic diagnosis | 16 | 1658 | 104 (114) | 52 (92) | 467, 21 | 446 |
| Medical imaging process | 24 | 2690 | 112 (118) | 67 (113) | 446, 12 | 434 |
| Medical staff (endoscopy and functional exploration room) | 13 | 318 | 24 (21) | 21 (19) | 83, 4 | 79 |
| Medical staff (ER) | 19 | 478 | 25 (28) | 12 (21) | 109, 1 | 108 |
| Medical staff (general) | 43 | 25,835 | 601 (605) | 348 (831) | 2,559, 33 | 2526 |
| Medical staff (operating room) | 4 | 145 | 36 (13) | 41 (11) | 47, 17 | 31 |
| Medical, diagnostic and therapeutic services of third parties | 4 | 156 | 39 (34) | 34 (50) | 79, 8 | 72 |
| Non-medical therapies and counselling | 2 | 107 | 53 (47) | 53 (33) | 87, 20 | 66 |
| Nuclear medicine and radiation oncology | 3 | 1215 | 405 (412) | 374 (411) | 832, 9 | 823 |
| Occupational therapy | 1 | 9 | 9 (–) | 9 (–) | 9, 9 | 0 |
| Operating rooms | 1 | 182 | 182 (–) | 182 (–) | 182, 182 | 0 |
| Pathology | 22 | 1687 | 77 (121) | 33 (44) | 520, 2 | 518 |
| Patient administration | 43 | 1428 | 33 (6) | 35 (8) | 42, 17 | 26 |
| Patient transport provided by third parties | 1 | 7 | 7 (–) | 7 (–) | 7, 7 | 0 |
| Physicians' fees, hospital doctors | 35 | 2,955 | 84 (103) | 51 (94) | 485, 1 | 483 |
| Physiotherapy | 2 | 216 | 108 (74) | 108 (53) | 160, 55 | 105 |
| Psychology | 1 | 64 | 64 (–) | 64 (–) | 64, 64 | 0 |
| *IQR = inter-quartile range; OI = Originator infliximab; sd = standard deviation* | | | | | | |

**Table VII** Details on mean and median outpatient costs by cost accounts for the cohort “Switched from OI to CT-P13 and maintained CT-P13”

| Cost account | Patients (n) | Total [CHF] | Mean (sd) [CHF] | Median (IQR) [CHF] | max, min [CHF] | Range [CHF] |
| --- | --- | --- | --- | --- | --- | --- |
| Accommodation (room) | 6 | 40 | 7 (11) | 3 (2) | 29, 1 | 28 |
| Anaesthesia | 3 | 307 | 102 (139) | 25 (122) | 263, 20 | 243 |
| Cardiac catheterisation laboratory | 1 | 31 | 31 (–) | 31 (–) | 31, 31 | 0 |
| Care | 54 | 23682 | 439 (403) | 302 (470) | 1859, 17 | 1843 |
| Drugs | 53 | 241440 | 4555 (4897) | 2761 (5384) | 21658, 4 | 21654 |
| Emergency room (ER) | 13 | 1097 | 84 (54) | 84 (85) | 188, 12 | 176 |
| Equipment, instruments, utensils, textiles | 53 | 3690 | 70 (234) | 15 (24) | 1601, 1 | 1601 |
| Laboratory | 54 | 11456 | 212 (172) | 135 (171) | 686, 18 | 668 |
| Medical and therapeutic diagnosis | 14 | 947 | 68 (68) | 38 (60) | 223, 14 | 209 |
| Medical imaging process | 29 | 3231 | 111 (125) | 56 (119) | 518, 4 | 513 |
| Medical staff (cardiac ward) | 1 | 5 | 5 (–) | 5 (–) | 5, 5 | 0 |
| Medical staff (endoscopy and functional exploration room) | 13 | 282 | 22 (16) | 24 (21) | 57, 4 | 53 |
| Medical staff (ER) | 13 | 321 | 25 (15) | 26 (26) | 48, 4 | 44 |
| Medical staff (general) | 54 | 28604 | 530 (848) | 181 (330) | 3739, 20 | 3719 |
| Medical staff (operating room) | 5 | 133 | 27 (34) | 13 (7) | 87, 4 | 83 |
| Medical, diagnostic and therapeutic services of third parties | 4 | 64 | 16 (23) | 7 (18) | 49, 0 | 49 |
| Non-medical therapies and counselling | 2 | 109 | 55 (57) | 55 (41) | 95, 14 | 81 |
| Nuclear medicine and radiation oncology | 3 | 219 | 73 (91) | 34 (84) | 177, 8 | 169 |
| Pathology | 17 | 923 | 54 (64) | 31 (47) | 230, 5 | 225 |
| Patient administration | 54 | 1927 | 36 (5) | 38 (4) | 47, 18 | 29 |
| Physicians' fees, hospital doctors | 50 | 3598 | 72 (67) | 58 (66) | 373, 3 | 369 |
| Physiotherapy | 1 | 61 | 61 (–) | 61 (–) | 61, 61 | 0 |
| *IQR = inter-quartile range; OI = Originator infliximab; sd = standard deviation* | | | | | | |

**Table VIII** Details on mean and median outpatient costs by cost accounts for the cohort “Switched from OI to CT-P13 and discontinued CT-P13”

| Cost account | Patients (n) | Total [CHF] | Mean (sd) [CHF] | Median (IQR) [CHF] | max, min [CHF] | Range [CHF] |
| --- | --- | --- | --- | --- | --- | --- |
| Accommodation (room) | 2 | 2 | 1 (1) | 1 (1) | 2, 1 | 1 |
| Anaesthesia | 2 | 54 | 27 (26) | 27 (18) | 45, 9 | 36 |
| Care | 16 | 4067 | 254 (196) | 177 (123) | 828, 72 | 756 |
| Delivery room | 1 | 17 | 17 (–) | 17 (–) | 17, 17 | 0 |
| Drugs | 16 | 33601 | 2100 (1416) | 1818 (2151) | 4592, 51 | 4542 |
| Emergency room (ER) | 8 | 307 | 38 (23) | 43 (38) | 68, 10 | 58 |
| Equipment, instruments, utensils, textiles | 16 | 299 | 19 (23) | 10 (13) | 91, 2 | 89 |
| Laboratory | 16 | 2867 | 179 (132) | 112 (222) | 422, 35 | 387 |
| Medical and therapeutic diagnosis | 8 | 537 | 67 (46) | 56 (72) | 133, 15 | 119 |
| Medical imaging process | 11 | 1034 | 94 (100) | 81 (71) | 365, 4 | 361 |
| Medical staff (endoscopy and functional exploration room) | 8 | 356 | 45 (36) | 42 (63) | 102, 6 | 97 |
| Medical staff (ER) | 7 | 138 | 20 (12) | 19 (14) | 37, 3 | 33 |
| Medical staff (general) | 16 | 7705 | 482 (485) | 298 (378) | 1802, 63 | 1740 |
| Medical staff (operating room) | 1 | 6 | 6 (–) | 6 (–) | 6, 6 | 0 |
| Medical, diagnostic and therapeutic services of third parties | 2 | 11 | 5 (–) | 5 (–) | 6, 6 | 0 |
| Nuclear medicine and radiation oncology | 2 | 846 | 423 (35) | 423 (25) | 448, 398 | 49 |
| Pathology | 8 | 519 | 65 (69) | 40 (57) | 188, 12 | 176 |
| Patient administration | 16 | 556 | 35 (5) | 36 (6) | 40, 24 | 16 |
| Physicians' fees, hospital doctors | 16 | 1516 | 95 (79) | 85 (112) | 233, 3 | 231 |
| Physiotherapy | 1 | 78 | 78 (–) | 78 (–) | 78, 78 | 0 |
| *IQR = inter-quartile range; OI = Originator infliximab; sd = standard deviation* | | | | | | |

**Table IX** Details on mean and median outpatient costs by cost accounts for the cohort “Switched from OI to CT-P13 and switched back to OI”

| Cost account | Patients (n) | Total [CHF] | Mean (sd) [CHF] | Median (IQR) [CHF] | max, min [CHF] | Range [CHF] |
| --- | --- | --- | --- | --- | --- | --- |
| Accommodation (room) | 4 | 3 | 1 (–) | 1 (–) | 1, | 1 |
| Anaesthesia | 1 | 392 | 392 (–) | 392 (–) | 392, 392 | 0 |
| Care | 14 | 6145 | 439 (322) | 280 (480) | 1089, 78 | 1011 |
| Drugs | 14 | 98828 | 7059 (5819) | 6082 (8087) | 16975, 1 | 16973 |
| Emergency room (ER) | 6 | 275 | 46 (34) | 40 (44) | 94, 4 | 90 |
| Equipment, instruments, utensils, textiles | 13 | 228 | 18 (17) | 13 (12) | 63, 1 | 62 |
| Laboratory | 16 | 3496 | 219 (176) | 141 (241) | 691, 5 | 687 |
| Medical and therapeutic diagnosis | 3 | 139 | 46 (34) | 34 (33) | 85, 20 | 65 |
| Medical imaging process | 6 | 689 | 115 (90) | 123 (139) | 234, 14 | 219 |
| Medical staff (endoscopy and functional exploration room) | 3 | 91 | 30 (30) | 16 (27) | 65, 10 | 54 |
| Medical staff (ER) | 4 | 59 | 15 (14) | 11 (14) | 34, 3 | 31 |
| Medical staff (general) | 15 | 4696 | 313 (338) | 228 (182) | 1423, 50 | 1373 |
| Medical staff (operating room) | 1 | 61 | 61 (–) | 61 (–) | 61, 61 | 0 |
| Medical, diagnostic and therapeutic services of third parties | 1 | 39 | 39 (–) | 39 (–) | 39, 39 | 0 |
| Non-medical therapies and counselling | 2 | 48 | 24 (4) | 24 (3) | 27, 21 | 6 |
| Nuclear medicine and radiation oncology | 1 | 300 | 300 (–) | 300 (–) | 300, 300 | 0 |
| Pathology | 6 | 223 | 37 (56) | 14 (11) | 150, 10 | 140 |
| Patient administration | 16 | 461 | 29 (9) | 31 (8) | 38, 6 | 32 |
| Physicians' fees, hospital doctors | 14 | 1032 | 74 (57) | 61 (75) | 178, 10 | 168 |
| Physiotherapy | 2 | 540 | 270 (169) | 270 (119) | 389, 151 | 239 |
| *IQR = inter-quartile range; OI = Originator infliximab; sd = standard deviation* | | | | | | |

### Comparison of means by bootstrap analysis

**Table X** Bootstrapped percentile intervals for mean outpatient costs, by cost account and by cohort

|  | Cost mean PCI [CHF] | | | |
| --- | --- | --- | --- | --- |
| **Cost account** | **Started CT-P13 and maintained CT-P13** | **Switched from OI to CT-P13 and maintained CT-P13** | **Switched from OI to CT-P13 and discontinued CT-P13** | **Switched from OI to CT-P13 and switched back to OI** |
| Accommodation (room) | (2.29 ; 26.08) | (1.711 ; 15.773) | (0.456 ; 1.566) | (0.5827 ; 1.0174) |
| Anaesthesia | (32.34 ; 185.32) | (19.8 ; 262.8) | (8.79 ; 45.27) | 1 patient |
| Care | (255.7 ; 420.5) | (335.7 ; 550.5) | (169.9 ; 358.9) | (288.1 ; 610.1) |
| Drugs | (1146 ; 2551) | (3339 ; 5918) | (1438 ; 2809) | (4176 ; 10086) |
| Emergency room (ER) | (42 ; 110.62) | (57.06 ; 112.32) | (22.95 ; 53.48) | (21.76 ; 70.42) |
| Equipment, instruments, utensils, textiles | (17.84 ; 35.33) | (24.05 ; 141.73) | (9.42 ; 30.66) | (9.76 ; 27.46) |
| Laboratory | (157.3 ; 239.9) | (168.2 ; 260.6) | (119.2 ; 242.8) | (141.4 ; 306.8) |
| Medical and therapeutic diagnosis | (57.2 ; 164.8) | (36.84 ; 104.29) | (37.34 ; 98.5) | (19.66 ; 84.92) |
| Medical imaging process | (70 ; 160.2) | (71.3 ; 158.5) | (45.98 ; 156.3) | (52.1 ; 182.5) |
| Medical staff (endoscopy and functional exploration room) | (15.04 ; 36.81) | (13.52 ; 30.72) | (23.65 ; 68.53) | (10.48 ; 64.9) |
| Medical staff (ER) | (14.04 ; 38.53) | (17.03 ; 32.38) | (11.83 ; 27.92) | (4.86 ; 26.97) |
| Medical staff (general) | (434 ; 788.8) | (318.8 ; 774.3) | (276.2 ; 734.4) | (180.8 ; 506) |
| Medical staff (operating room) | (23.22 ; 45.18) | (8.14 ; 57.55) | 1 patient | 1 patient |
| Medical, diagnostic and therapeutic services of third parties | (10.37 ; 67.51) | (1.76 ; 37.76) | (5.146 ; 5.62) | 1 patient |
| Non-medical therapies and counselling | (20.41 ; 86.55) | (13.95 ; 95.1) | no patient | (21.18 ; 27.17) |
| Nuclear medicine and radiation oncology | (9.2 ; 831.8) | (8.19 ; 176.96) | (398.4 ; 447.5) | 1 patient |
| Pathology | (35.14 ; 132.39) | (28.15 ; 86.85) | (25.25 ; 116.31) | (11.64 ; 83.05) |
| Patient administration | (31.33 ; 34.9) | (34.4 ; 36.91) | (32.41 ; 36.85) | (24.41 ; 32.67) |
| Physicians' fees, hospital doctors | (53.66 ; 120.52) | (55.2 ; 91.42) | (58.36 ; 132) | (47.22 ; 103.49) |
| Physiotherapy | (55.3 ; 160.3) | 1 patient | 1 patient | (150.8 ; 389.4) |
| *PCI = percentile confidence intervals* | | | | |

### Multivariable analysis on costs by generalized linear model (GLM)

| Analysis of Deviance Table | Df.res | Dev | Df | Deviance | Pr(>Chi) | AIC | BIC |
| --- | --- | --- | --- | --- | --- | --- | --- |
| cost ~ 1 | 254 | 200.85 | — | — | — | 4856 | 4863 |
| cost ~ 1 + genre | 253 | 249.5 | 1 | 0.20 | 0.75 | 4858 | 4868 |
| cost ~ 1 + age | 253 | 249.54 | 1 | 0.16 | 0.78 | 4858 | 4868 |
| cost ~ 1 + disease_cat | 252 | 197.71 | 2 | 51.99 | 1.39E-08 | 4792 | 4806 |
| *AIC = Akaike information criterion; BIC = Bayesian information criterion; Dev = Deviance; Deviance = Difference in deviance; Df = Degrees of freedom; Df.res = Residual degrees of freedom; Pr(>Chi) = p value from likelihood ratio test;* | | | | | | | |

**Table XI** Likelihood ratio test and variable estimates of the model using disease category as an independent variable

| *p*-value (LR test) and estimates | Gastroenterology | Immunoallergology |
| --- | --- | --- |
| Gastroenterology | — | 0.71 (-0.07) |
| Rheumatology | **9.69E-08** (0.95) | **9.69E-05** (0.88) |
| *LR test = likelihood ratio test* | | |

## Inpatient costs

**Table XII** Details on inpatient costs and comparison of variances and means by cohort

| Patients  (n) | Total  [CHF] | Mean (sd)  [CHF] | Median (IQR)  [CHF] | max, min  [CHF] | Range  [CHF] | Bartlett  [*p* value] | Levene  [*p* value] | Fligner  [*p* value] | ANOVA  [*p* value] | ANOVA_u  [*p* value] |
| --- | --- | --- | --- | --- | --- | --- | --- | --- | --- | --- |
| 94 | CHF 151 465 | CHF 1,611 (1,020) | CHF 1 437 ( 995) | CHF 5 057, CHF 127 | CHF 4 930 | **0.01** | 0.07 | 0.10 | 0.06 | 0.20 |
| *ANOVA = Analysis of variance; ANOVA_U = One-way ANOVA or analysis of variance for samples whose variances are not necessarily assumed to be equal; Bartlett = Bartlett's test; Fligner = Fligner-Killeen test; IQR = inter-quartile range; k = one thousand; Levene = Levene's test; sd = standard deviation* | | | | | | | | | | |

**Table XIII** Details on mean and median inpatient costs by cohort, normality assessment of the data was performed using the Shapiro-Wilk normality test and the Kolmogorov-Smirnov test for samples with *n* < 50 patients and *n* > 50 patients, respectively.

| Cohort | patients  (n) | Total  [CHF] | Mean (sd)  [CHF] | Median (IQR)  [CHF] | max, min  [CHF] | Range  [CHF] | Shapiro  [*p* value] | Kolmogorov  [*p* value] |
| --- | --- | --- | --- | --- | --- | --- | --- | --- |
| Started OI and maintained OI | 27 | 54 889 | 2,033 (1,096) | 1 658 (1 090) | 5 057, 352 | 4 705 | **0.02** | — |
| Started CT-P13 and maintained CT-P13 | 22 | 32 044 | 1,457 (,747) | 1 247 ( 732) | 3 799, 517 | 3 282 | **2.63E-03** | — |
| Started OI and discontinued OI | 6 | 8 636 | 1,439 (,453) | 1 488 ( 640) | 1 899, 770 | 1 129 | 0.39 | — |
| Started CT-P13 and discontinued CT-P13 | 21 | 26 607 | 1,267 (,767) | 1 205 ( 788) | 3 674, 145 | 3 528 | **0.03** | — |
| Switched from OI to CT-P13 and maintained CT-P13 | 8 | 13 815 | 1,727 (1,748) | 973 (2 714) | 4 707, 206 | 4 502 | 0.08 | — |
| Switched from OI to CT-P13 and discontinued CT-P13 | 7 | 7 935 | 1,134 (,855) | 926 (1 126) | 2 542, 127 | 2 415 | 0.71 | — |
| Switched from OI to CT-P13 and switched back to OI | 3 | 7 538 | 2,513 (1,236) | 2 086 (1 179) | 3 905, 1 547 | 2 359 | 0.42 | — |
| *IQR = inter-quartile range; Kolmogorov = Asymptotic one-sample Kolmogorov-Smirnov test; OI = Originator infliximab; sd = standard deviation; Shapiro = Shapiro-Wilk normality test* | | | | | | | | |

### Normality tests and plots

#### Shapiro-Wilk normality test for all included patients (n samples < 50) - for information purposes only.

W = 0.9049, *p*-value = 4.453e-06 🡪 *inpatient cost* does not follow a normal distribution

#### Asymptotic one-sample Kolmogorov-Smirnov test all included patients (*n* samples ≥ 50) :

D = 0.80027, p-value = < 2.2e-16 🡪 *inpatient cost* does not follow a normal distribution


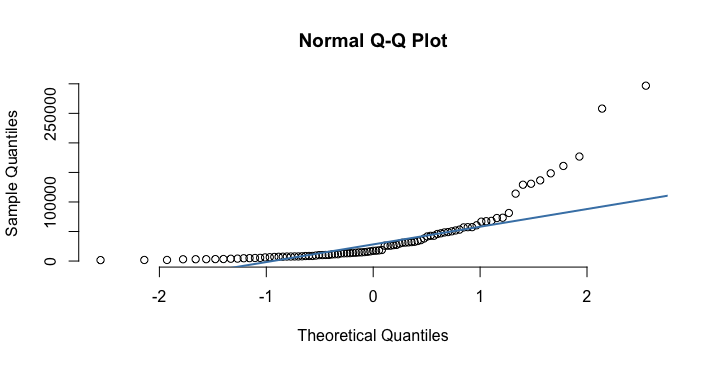


**Figure IX.** Quantile-Quantile plot of inpatient costs (left) (*n* = 94)

**Figure X.** Box plot of inpatient costs (center) (*n* = 94)

**Figure XI.** Density plot of inpatient costs (right) (*n* = 94)

**Figure XII.** Density plots of inpatient costs, by cohort

### Comparison of means by bootstrap analysis

**Table XIV** Bootstrapped percentile intervals for mean inpatient costs, by cohort

| Cohort | Bootstrap intervals [CHF] |
| --- | --- |
| Started OI and maintained OI | (1 641 ; 2 440) |
| Started CT-P13 and maintained CT-P13 | (1 176 ; 1 781) |
| Started OI and discontinued OI | (1 088 ; 1 743) |
| Started CT-P13 and discontinued CT-P13 | ( 968 ; 1 612) |
| Switched from OI to CT-P13 and maintained CT-P13 | ( 671 ; 2 917) |
| Switched from OI to CT-P13 and discontinued CT-P13 | ( 585 ; 1 764) |
| Switched from OI to CT-P13 and switched back to OI | (1 547 ; 3 905) |
| *OI = Originator infliximab* | |

### Inpatient costs per cost account

**Table XV** Details on mean and median inpatient costs by cost accounts for the cohort “Started CT-P13 and discontinued CT-P13”

| Cost account | Patients (n) | Total [CHF] | Mean (sd) [CHF] | Median (IQR) [CHF] | max, min [CHF] | Range [CHF] |
| --- | --- | --- | --- | --- | --- | --- |
| Accommodation (kitchen) | 21 | 1236 | 59 (28) | 65 (37) | 106, 7 | 99 |
| Accommodation (room) | 21 | 1520 | 72 (43) | 71 (67) | 153, 9 | 144 |
| Anaesthesia | 7 | 530 | 76 (33) | 70 (36) | 141, 48 | 92 |
| Blood and blood products | 2 | 249 | 125 (154) | 125 (109) | 233, 16 | 217 |
| Care | 21 | 7243 | 345 (244) | 305 (298) | 849, | 849 |
| Delivery room | 1 | 38 | 38 (–) | 38 (–) | 38, 38 | 0 |
| Drugs | 20 | 686 | 34 (48) | 17 (22) | 175, 0 | 175 |
| Emergency room (ER) | 12 | 735 | 61 (43) | 56 (57) | 137, 11 | 126 |
| Equipment, instruments, utensils, textiles | 18 | 566 | 31 (47) | 8 (39) | 164, 1 | 163 |
| Intensive care units (ICU) and intermediate care units (IMCU) | 6 | 706 | 118 (129) | 67 (118) | 354, 17 | 337 |
| Intermediate Care Units (IMCU) | 20 | 2588 | 129 (201) | 65 (88) | 941, 3 | 938 |
| Laboratory | 9 | 457 | 51 (79) | 16 (41) | 252, 6 | 247 |
| Medical and therapeutic diagnosis | 16 | 802 | 50 (63) | 22 (36) | 232, 6 | 226 |
| Medical imaging process | 1 | 10 | 10 (–) | 10 (–) | 10, 10 | 0 |
| Medical staff (delivery room) | 9 | 143 | 16 (18) | 6 (14) | 47, 2 | 45 |
| Medical staff (endoscopy and functional exploration room) | 10 | 245 | 24 (16) | 23 (27) | 49, 2 | 46 |
| Medical staff (ER) | 21 | 5252 | 250 (206) | 194 (185) | 977, 7 | 970 |
| Medical staff (general) | 6 | 127 | 21 (14) | 18 (14) | 43, 3 | 40 |
| Medical staff (ICU) | 7 | 334 | 48 (22) | 54 (30) | 76, 15 | 60 |
| Medical staff (IMCU) | 12 | 235 | 20 (20) | 10 (20) | 61, 2 | 59 |
| Medical staff (operating room) | 18 | 126 | 7 (10) | 3 (8) | 36, 0 | 36 |
| Medical, diagnostic and therapeutic services of third parties | 3 | 17 | 6 (6) | 2 (5) | 13, 2 | 11 |
| Non-medical therapies and counselling | 7 | 944 | 135 (56) | 126 (65) | 226, 69 | 157 |
| Nuclear medicine and radiation oncology | 21 | 25 | 1 (1) | 1 (1) | 2, 0 | 2 |
| Occupational therapy | 12 | 687 | 57 (97) | 26 (27) | 359, 8 | 350 |
| Operating rooms | 21 | 948 | 45 (44) | 29 (28) | 173, 8 | 165 |
| Other service providers | 9 | 159 | 18 (19) | 7 (21) | 58, 2 | 56 |
| Pathology | 21 | 1236 | 59 (28) | 65 (37) | 106, 7 | 99 |
| Patient administration | 21 | 1520 | 72 (43) | 71 (67) | 153, 9 | 144 |
| Patient transport provided by third parties | 7 | 530 | 76 (33) | 70 (36) | 141, 48 | 92 |
| Physiotherapy | 2 | 249 | 125 (154) | 125 (109) | 233, 16 | 217 |
| Speech therapy | 21 | 7243 | 345 (244) | 305 (298) | 849, 0 | 849 |
| *IQR = inter-quartile range; OI = Originator infliximab; sd = standard deviation* | | | | | | |

**Table XVI** Details on mean and median inpatient costs by cost accounts for the cohort “Started OI and maintained OI”

| Cost account | Patients (n) | Total [CHF] | Mean (sd) [CHF] | Median (IQR) [CHF] | max, min [CHF] | Range [CHF] |
| --- | --- | --- | --- | --- | --- | --- |
| Accommodation (kitchen) | 27 | 1932 | 72 (25) | 75 (32) | 103, 8 | 96 |
| Accommodation (room) | 27 | 2752 | 102 (56) | 98 (47) | 247, 7 | 239 |
| Anaesthesia | 11 | 2133 | 194 (204) | 90 (278) | 583, 16 | 566 |
| Blood and blood products | 1 | 5 | 5 (–) | 5 (–) | 5, 5 | 0 |
| Care | 27 | 10414 | 386 (209) | 360 (168) | 1145, 66 | 1079 |
| Delivery room | 1 | 158 | 158 (–) | 158 (–) | 158, 158 | 0 |
| Drugs | 27 | 1225 | 45 (114) | 10 (36) | 586, | 586 |
| Emergency room (ER) | 15 | 2056 | 137 (206) | 62 (83) | 797, 14 | 783 |
| Equipment, instruments, utensils, textiles | 23 | 4579 | 199 (427) | 15 (51) | 1447, 2 | 1446 |
| Intensive care units (ICU) and intermediate care units (IMCU) | 2 | 914 | 457 (288) | 457 (204) | 661, 253 | 408 |
| Intermediate Care Units (IMCU) | 9 | 1082 | 120 (91) | 136 (160) | 267, 1 | 266 |
| Laboratory | 26 | 3270 | 126 (145) | 86 (123) | 681, 3 | 678 |
| Medical and therapeutic diagnosis | 17 | 2110 | 124 (211) | 28 (121) | 795, 4 | 791 |
| Medical imaging process | 18 | 1589 | 88 (67) | 61 (91) | 246, 8 | 238 |
| Medical staff (delivery room) | 2 | 29 | 15 (4) | 15 (3) | 17, 12 | 5 |
| Medical staff (endoscopy and functional exploration room) | 15 | 720 | 48 (54) | 26 (30) | 180, 2 | 178 |
| Medical staff (ER) | 15 | 768 | 51 (51) | 34 (53) | 191, 0 | 191 |
| Medical staff (general) | 27 | 7970 | 295 (250) | 238 (175) | 1343, 20 | 1322 |
| Medical staff (ICU) | 3 | 609 | 203 (237) | 104 (221) | 473, 32 | 442 |
| Medical staff (IMCU) | 8 | 463 | 58 (37) | 81 (54) | 87, 0 | 87 |
| Medical staff (operating room) | 9 | 391 | 43 (45) | 28 (65) | 118, 2 | 116 |
| Medical, diagnostic and therapeutic services of third parties | 14 | 241 | 17 (16) | 13 (13) | 54, 3 | 51 |
| Non-medical therapies and counselling | 21 | 105 | 5 (8) | 2 (1) | 36, 0 | 36 |
| Nuclear medicine and radiation oncology | 6 | 2876 | 479 (344) | 448 (536) | 933, 100 | 833 |
| Occupational therapy | 4 | 161 | 40 (40) | 30 (44) | 94, 6 | 88 |
| Operating rooms | 9 | 2943 | 327 (439) | 125 (355) | 1408, 29 | 1379 |
| Other service providers | 27 | 43 | 2 (–) | 2 (–) | 2, 0 | 2 |
| Pathology | 11 | 870 | 79 (145) | 21 (16) | 484, 10 | 474 |
| Patient administration | 27 | 1670 | 62 (41) | 40 (47) | 159, 14 | 144 |
| Patient transport provided by third parties | 2 | 100 | 50 (20) | 50 (14) | 64, 36 | 28 |
| Physiotherapy | 11 | 583 | 53 (62) | 33 (68) | 208, 1 | 208 |
| Speech therapy | 2 | 126 | 63 (71) | 63 (50) | 113, 13 | 100 |
| *IQR = inter-quartile range; OI = Originator infliximab; sd = standard deviation* | | | | | | |

#### Comparison of means by bootstrap analysis

**Table XVII** Bootstrapped percentile intervals for mean inpatient costs, by cost account and by cohort

|  | Cost mean PCI [CHF] | |
| --- | --- | --- |
| **Cost account** | **Started CT-P13 and discontinued CT-P13** | **Started OI and maintained OI** |
| Accommodation (kitchen) | (46.73 ; 70.33) | (62.14 ; 80.11) |
| Accommodation (room) | (54.87 ; 90.54) | (81.8 ; 123.4) |
| Anaesthesia | (55.71 ; 101.54) | (90 ; 316.9) |
| Care | (246.2 ; 451.9) | (314.2 ; 466.5) |
| Drugs | (15.92 ; 57.45) | (15.74 ; 94.09) |
| Emergency room (ER) | (39.86 ; 85.04) | (57.9 ; 250.2) |
| Equipment, instruments, utensils, textiles | (12.4 ; 55.17) | (48.9 ; 389.8) |
| Intermediate Care Units (IMCU) | (38.3 ; 219.4) | (61.6 ; 177.1) |
| Laboratory | (67 ; 226.2) | (76.8 ; 187.5) |
| Medical and therapeutic diagnosis | (15.33 ; 105.29) | (41.1 ; 236.2) |
| Medical imaging process | (23.79 ; 84.61) | (59.87 ; 119.83) |
| Medical staff (endoscopy and functional exploration room) | (5.77 ; 28.59) | (24.81 ; 76.49) |
| Medical staff (ER) | (15.51 ; 33.56) | (28.77 ; 78.89) |
| Medical staff (general) | (176.3 ; 347.1) | (214.1 ; 400.2) |
| Medical staff (IMCU) | (11.34 ; 32.22) | (32.41 ; 79.61) |
| Medical staff (operating room) | (32.41 ; 62.15) | (16.91 ; 72.9) |
| Medical, diagnostic and therapeutic services of third parties | (9.59 ; 31.5) | (10.01 ; 26.13) |
| Non-medical therapies and counselling | (3.156 ; 11.849) | (2.066 ; 8.888) |
| Occupational therapy | (1.838 ; 12.805) | (10.69 ; 74.6) |
| Operating rooms | (98.5 ; 175.4) | (114.4 ; 620.9) |
| Other service providers | (0.936 ; 1.435) | (1.404 ; 1.76) |
| Pathology | (21.15 ; 116.81) | (18.63 ; 166.54) |
| Patient administration | (28.68 ; 65.71) | (47.13 ; 78.01) |
| Physiotherapy | (6.83 ; 31.37) | (23.24 ; 91.55) |
| *ER = Emergency room ; PCI = Percentile confidence intervals* | | |

### Multivariable analysis on costs by generalized linear model (GLM)

**Table XVIII** Nested GLMs along with their parameters

| Analysis of Deviance Table | Df.res | Dev | Df | Deviance | Pr(>Chi) | AIC | BIC |
| --- | --- | --- | --- | --- | --- | --- | --- |
| cost ~ 1 | 93 | 41 |  |  |  | 1 547 | 1 552 |
| cost ~ 1 + genre | 92 | 41 | 1 | 0.15 | 0.54 | 1 549 | 1 556 |
| cost ~ 1 + age | 92 | 37 | 1 | 4.19 | **6.56E-04** | 1 538 | 1 546 |
| cost ~ 1 + disease_cat | 91 | 32 | 2 | 9.45 | **5.68E-07** | 1 525 | 1 535 |
| *AIC = Akaike information criterion; BIC = Bayesian information criterion; Dev = Deviance; Deviance = Difference in deviance; Df = Degrees of freedom; Df.res = Residual degrees of freedom; Pr(>Chi) = p value from likelihood ratio test* | | | | | | | |

**Figure XIII.** (**A**) Scatterplot of inpatient cost per hospital day by age and (**B**) by disease categories.

**Table XIX** Likelihood ratio test and variable estimates of the model using disease category as an independent variable

| *p*-value (LR test) and estimates | Gastroenterology | Immunoallergology |
| --- | --- | --- |
| Gastroenterology | — | **1.09E-06** (-0.69) |
| Rheumatology | **3.37E-03** (0.49) | 0.24 (-0.20) |
| *LR test = likelihood ratio test* | | |

## Length of stay (LOS)

**Table XX** Details on length of stay of inpatients and comparison of variances, means and medians by cohort

| Patients  (n) | Mean (sd)  [days] | Median (IQR)  [days] | max, min  [days] | Range  [days] | Bartlett  [*p* value] | Levene  [*p* value] | Fligner  [*p* value] | ANOVA  [*p* value] | ANOVA_u  [*p* value] | Kruskal  [*p* value] |
| --- | --- | --- | --- | --- | --- | --- | --- | --- | --- | --- |
| 94 | 20 (28) | 24 (10) | 187, 1 | 186 | **2.07E-08** | **< 2.2e-16** | **0.02** | **0.01** | 0.08 | **0.01** |
| *ANOVA = Analysis of variance; ANOVA_U = One-way ANOVA or analysis of variance for samples whose variances are not necessarily assumed to be equal; Bartlett = Bartlett's test; Fligner = Fligner-Killeen test; IQR = inter-quartile range; Kruskal = Kruskal–Wallis test; Levene = Levene's test; sd = standard deviation* | | | | | | | | | | |

**Table XXI** Details on mean and median of length of stay by cohort, and normality assessment of the data

| Cohort | patients (n) | Mean (sd)  [days] | Median (IQR)  [days] | max, min  [days] | Range  [days] | Shapiro  [*p* value] | |
| --- | --- | --- | --- | --- | --- | --- | --- |
| Started OI and maintained OI | 27 | 12 (13) | 7 (49) | 49, 1 | 48 | **4.16E-05** | |
| Started CT-P13 and maintained CT-P13 | 22 | 31 (41) | 21 (187) | 187, 1 | 186 | **2.83E-06** | |
| Started OI and discontinued OI | 6 | 8 (5) | 8 (14) | 14, 2 | 12 | 0.29 | |
| Started CT-P13 and discontinued CT-P13 | 21 | 23 (26) | 15 (101) | 101, 1 | 100 | **6.95E-04** | |
| Switched from OI to CT-P13 and maintained CT-P13 | 8 | 8 (13) | 2 (39) | 39, 1 | 38 | **5.14E-05** | |
| Switched from OI to CT-P13 and discontinued CT-P13 | 7 | 12 (12) | 6 (32) | 32, 1 | 31 | 0.09 | |
| Switched from OI to CT-P13 and switched back to OI | 3 | 62 (42) | 76 (95) | 95, 15 | 80 | 0.44 | |
| *IQR = inter-quartile range; Kolmogorov = Asymptotic one-sample Kolmogorov-Smirnov test; OI = Originator infliximab; sd = standard deviation; Shapiro = Shapiro-Wilk normality test* | | | | | | |  |

### Normality tests and plots

#### Shapiro-Wilk normality test for all included patients (n samples < 50) - for information purposes only.

W = 0.64835, p-value = 1.124e-13 🡪 *LOS* does not follow a normal distribution

#### Asymptotic one-sample Kolmogorov-Smirnov test all included patients (*n* samples ≥ 50) :

D = 0.24894, p-value = 1.743e-05🡪 *LOS* does not follow a normal distribution


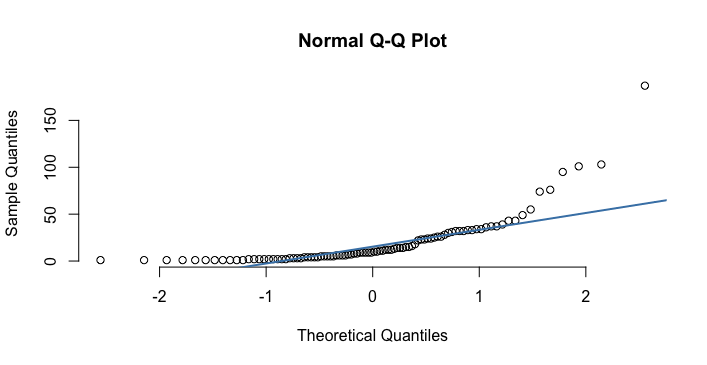


**Figure XIV.** Quantile-Quantile plot of inpatient length of stay (left) (*n* = 94)

**Figure XV.** Box plot of inpatient length of stay (center) (*n* = 94)

**Figure XVI.** Box plots of inpatient length of stay, by cohort

**Figure XVII.** Density plots of inpatient length of stay, by cohort

### Comparison of means by bootstrap analysis

**Table XXII** Bootstrapped percentile intervals for mean inpatient length of stay of, by cohort

| Cohort | Bootstrapped mean intervals [days] |
| --- | --- |
| Started OI and maintained OI | (7.67 ; 17.26) |
| Started CT-P13 and maintained CT-P13 | (16.86 ; 50.14) |
| Started OI and discontinued OI | (4.00 ; 11.67) |
| Started CT-P13 and discontinued CT-P13 | (13.48 ; 34.86) |
| Switched from OI to CT-P13 and maintained CT-P13 | (2.00 ; 17.00) |
| Switched from OI to CT-P13 and discontinued CT-P13 | (3.71 ; 21.00) |
| Switched from OI to CT-P13 and switched back to OI | (15.00 ; 95.00) |
| *OI = originator infliximab* | |

### Multivariable analysis on costs by generalized linear model (GLM)

**Table XXIII** Nested GLMs along with their parameters

| Analysis of Deviance Table | Df.res | Dev | Df | Deviance | Pr(>Chi) | AIC | BIC |
| --- | --- | --- | --- | --- | --- | --- | --- |
| cost ~ 1 | 93 | 41 |  |  |  | 751 | 756 |
| cost ~ 1 + genre | 92 | 41 | 1 | 0.15 | 0.62 | 753 | 760 |
| cost ~ 1 + age | 92 | 37 | 1 | 4.19 | 0.15 | 749 | 757 |
| cost ~ 1 + disease_cat | 91 | 32 | 2 | 9.45 | 0.16 | 749 | 757 |
| *AIC = Akaike information criterion; BIC = Bayesian information criterion; Dev = Deviance; Deviance = Difference in deviance; Df = Degrees of freedom; Df.res = Residual degrees of freedom; Pr(>Chi) = p value from likelihood ratio test* | | | | | | | |

# Variable analysis

## Age

**Table XXIV** Details on age of all included patients and comparison of variances, means and medians by cohort

| patients (n) | Mean (sd)  [years] | Median (IQR)  [years] | max, min  [years] | Range  [years] | Bartlett  [*p* value] | Levene  [*p* value] | Fligner  [*p* value] | ANOVA  [*p* value] | ANOVA_u  [*p* value] | Kruskal  [*p* value] |
| --- | --- | --- | --- | --- | --- | --- | --- | --- | --- | --- |
| 258 | 41 (19) | 42 (28) | 88, 8 | 80 | 0.49 | 0.13 | 0.45 | **5.70E-03** | **1.78E-03** | **5.20E-03** |
| *ANOVA = Analysis of variance; ANOVA_U = One-way ANOVA or analysis of variance for samples whose variances are not necessarily assumed to be equal; Bartlett = Bartlett's test; Fligner = Fligner-Killeen test; IQR = inter-quartile range; Kruskal = Kruskal–Wallis test by ranks; Levene = Levene's test; sd = standard deviation* | | | | | | | | | | |

**Table XXV** Details on mean and median of age by cohort, and normality assessment of the data (*n* = 258)

| Cohort | patients (n) | Mean (sd)  [years] | Median (IQR)  [years] | max, min  [years] | Range  [years] | Shapiro  [*p* value] | Kolmogorov  [*p* value] |  |
| --- | --- | --- | --- | --- | --- | --- | --- | --- |
| Started OI and maintained OI | 78 | 43 (20) | 45 (30) | 88, 10 | 78 | — | 0.17 |  |
| Started CT-P13 and maintained CT-P13 | 43 | 39 (19) | 36 (25) | 84, 8 | 76 | 0.15 | — |  |
| Started OI and discontinued OI | 19 | 35 (14) | 34 (25) | 54, 13 | 42 | 0.12 | — |  |
| Started CT-P13 and discontinued CT-P13 | 32 | 34 (16) | 32 (19) | 69, 13 | 56 | 0.06 | — |  |
| Switched from OI to CT-P13 and maintained CT-P13 | 54 | 44 (20) | 48 (32) | 83, 12 | 71 | — | 0.46 |  |
| Switched from OI to CT-P13 and discontinued CT-P13 | 16 | 39 (18) | 39 (28) | 67, 14 | 54 | 0.32 | — |  |
| Switched from OI to CT-P13 and switched back to OI | 16 | 55 (16) | 54 (13) | 85, 13 | 72 | 0.25 | — |  |
| *IQR = inter-quartile range; Kolmogorov = Asymptotic one-sample Kolmogorov-Smirnov test; OI = Originator infliximab; sd = standard deviation; Shapiro = Shapiro-Wilk normality test* | | | | | | | | |

### Mean and median comparisons

**Table XXVI** Comparison of means with Student’s *t* test and Holm's post-hoc adjustment.

| Student's *t* test  [*p* value] | Started OI and maintained OI | Started CT-P13 and maintained CT-P13 | Started OI and discontinued OI | Started CT-P13 and discontinued CT-P13 | Switched from OI to CT-P13 and maintained CT-P13 | Switched from OI to CT-P13 and discontinued CT-P13 | |
| --- | --- | --- | --- | --- | --- | --- | --- |
| Started OI and maintained OI | — | — | — | — | — | — | |
| Started CT-P13 and maintained CT-P13 | 1.00 | — | — | — | — | — | |
| Started OI and discontinued OI | 0.72 | 1.00 | — | — | — | — | |
| Started CT-P13 and discontinued CT-P13 | 0.20 | 1.00 | 1.00 | — | — | — | |
| Switched from OI to CT-P13 and maintained CT-P13 | 1.00 | 1.00 | 0.64 | 0.19 | — | — | |
| Switched from OI to CT-P13 and discontinued CT-P13 | 1.00 | 1.00 | 1.00 | 1.00 | 1.00 | — | |
| Switched from OI to CT-P13 and switched back to OI | 0.20 | 0.06 | **0.01** | **2.70E-03** | 0.34 | 0.20 | |
| *OI = Originator infliximab* | | | | | | |  |

**Table XXVII** Comparison of medians with Wilcoxon’s rank sum test and Holm's post-hoc adjustment

| Wilcoxon's rank sum test [*p* value] | Started OI and maintained OI | Started CT-P13 and maintained CT-P13 | Started OI and discontinued OI | Started CT-P13 and discontinued CT-P13 | Switched from OI to CT-P13 and maintained CT-P13 | Switched from OI to CT-P13 and discontinued CT-P13 | |
| --- | --- | --- | --- | --- | --- | --- | --- |
| Started OI and maintained OI | — | — | — | — | — | — | |
| Started CT-P13 and maintained CT-P13 | 1.00 | — | — | — | — | — | |
| Started OI and discontinued OI | 1.00 | 1.00 | — | — | — | — | |
| Started CT-P13 and discontinued CT-P13 | 0.31 | 1.00 | 1.00 | — | — | — | |
| Switched from OI to CT-P13 and maintained CT-P13 | 1.00 | 1.00 | 1.00 | 0.30 | — | — | |
| Switched from OI to CT-P13 and discontinued CT-P13 | 1.00 | 1.00 | 1.00 | 1.00 | 1.00 | — | |
| Switched from OI to CT-P13 and switched back to OI | 0.26 | **0.04** | **0.01** | **1.10E-03** | 0.44 | 0.35 | |
| *OI = Originator infliximab* | | | | | | |  |

### Normality tests and plots

#### Shapiro-Wilk normality test for all included patients (n samples < 50) - for information purposes only.

W = 0.97355, p-value = 1.302e-05 🡪 *Age* does not follow a normal distribution

#### Asymptotic one-sample Kolmogorov-Smirnov test all included patients (*n* samples ≥ 50) :

D = 0.073445, p-value = 0.1236 🡪 *Age* follows a normal distribution


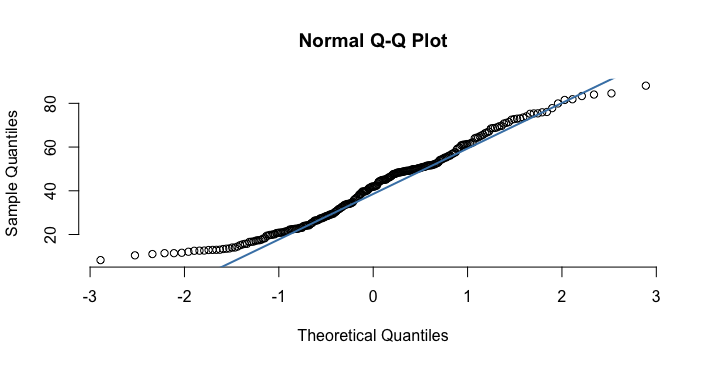

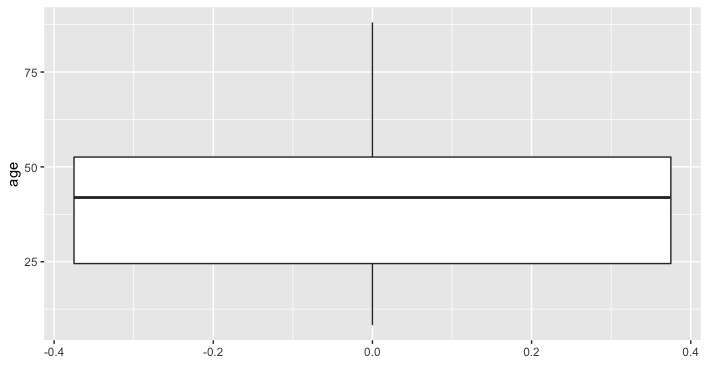

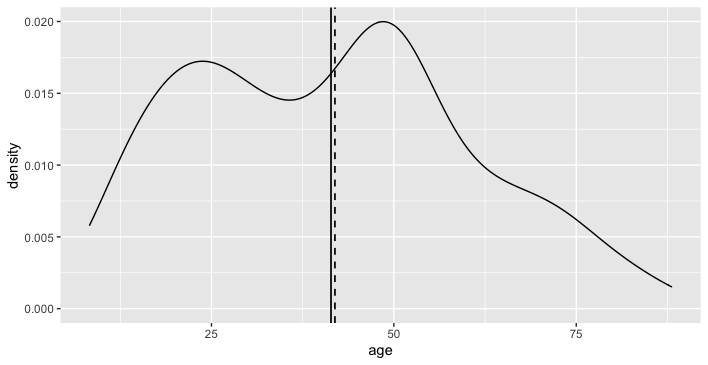


**Figure XVIII.** Quantile-Quantile plot of the age of the included patients (left) (*n* = 258)

**Figure XIX.** Box plot of the age of the included patients (center) (*n* = 258)

**Figure XX.** Density plot of the age of the included patients (right). The mean and the median are represented by the hard and dotted lines, respectively (*n* = 258)

**Figure XXI**. Quantile-Quantile plots of the age of the included patients, by cohort

**Figure XXII**. Box plots of the age of the included patients, by cohort

**Figure XXIII**. Density plots of the age of the included patients, by cohort

## Sex

**Table XXVIII** Comparison of sex proportions using the 2-sample test of equality of proportions and Yate's continuity correction for *n* < 5

| 2-sample test for equality of proportions (*p*-value) | Started oi and maintained oi | Started ct-p13 and maintained ct-p13 | Started oi and discontinued oi | Started ct-p13 and discontinued ct-p13 | Switched from oi to ct-p13 and maintained ct-p13 | Switched from oi to ct-p13 and discontinued ct-p13 |
| --- | --- | --- | --- | --- | --- | --- |
| Started OI and maintained OI | — | — | — | — | — | — |
| Started CT-P13 and maintained CT-P13 | 0.93 | — | — | — | — | — |
| Started OI and discontinued OI | 0.06 | 0.08 | — | — | — | — |
| Started CT-P13 and discontinued CT-P13 | 0.68 | 0.66 | 0.18 | — | — | — |
| Switched from OI to CT-P13 and maintained CT-P13 | 0.52 | 0.63 | **0.03** | 0.37 | — | — |
| Switched from OI to CT-P13 and discontinued CT-P13 | 0.89 | 0.94 | 0.15 | 0.68 | 0.79 | — |
| Switched from OI to CT-P13 and switched back to OI | 0.16 | 0.18 | 0.78 | 0.34 | 0.08 | 0.27 |

**Table XXIX** Comparison of sex proportions using the Fisher's Exact test for count data

| Fisher's Exact Test for Count Data (*p*-value) | Started OI and maintained OI | Started CT-P13 and maintained CT-P13 | Started OI and discontinued OI | Started CT-P13 and discontinued CT-P13 | Switched from OI to CT-P13 and maintained CT-P13 | Switched from OI to CT-P13 and discontinued CT-P13 |
| --- | --- | --- | --- | --- | --- | --- |
| Started OI and maintained OI | — | — | — | — | — | — |
| Started CT-P13 and maintained CT-P13 | 1.00 | — | — | — | — | — |
| Started OI and discontinued OI | **0.04** | 0.05 | — | — | — | — |
| Started CT-P13 and discontinued CT-P13 | 0.68 | 0.82 | 0.14 | — | — | — |
| Switched from OI to CT-P13 and maintained CT-P13 | 0.60 | 0.69 | **0.02** | 0.50 | — | — |
| Switched from OI to CT-P13 and discontinued CT-P13 | 1.00 | 1.00 | 0.09 | 0.76 | 1.00 | — |
| Switched from OI to CT-P13 and switched back to OI | 0.10 | 0.14 | 1.00 | 0.34 | 0.05 | 0.27 |

1. https://www.investing.com/currencies/chf-usd-historical-data [↑](#footnote-ref-1)
